# Mapping faculty development needs in medical education: a bibliometric analysis

**DOI:** 10.3389/fmed.2026.1858624

**Published:** 2026-07-01

**Authors:** Afaf Sulaiman Alblooshi, Farida Almarzooqi, Saif Al-Shamsi, Gamila Ahmed, Mona Hmoud Al-Sheikh

**Affiliations:** 1 Department of Medical Education, College of Medicine and Health Sciences, United Arab Emirates University, Al Ain/Abu Dhabi, United Arab Emirates; 2 Department of Internal Medicine, College of Medicine and Health Sciences, United Arab Emirates University, Al Ain/Abu Dhabi, United Arab Emirates; 3 Public Services and Outreach Unit, National Medical Library, College of Medicine and Health Sciences, United Arab Emirates University, Al Ain/Abu Dhabi, United Arab Emirates

**Keywords:** bibliometric analysis, competency-based medical education, faculty development, health professions education, medical education, needs assessment, professional development, research trends

## Abstract

**Background:**

Faculty development is central to sustaining and improving the quality of medical education; however, the cumulative scholarly landscape—its growth trajectories, geographic concentration, thematic architecture, and methodological foundations—has not been systematically mapped. This bibliometric analysis aims to characterize the intellectual structure of faculty development needs assessment research in medical education over a 20-year period (2005–2025) and to identify priority development needs consistently reported by faculty across diverse institutional contexts.

**Methods:**

Bibliometric analysis was conducted using Biblioshiny (Bibliometrix v5.1.0, R v4.4.1) on a deduplicated dataset from Web of Science, Scopus, and PubMed. Analyses included publication trends, geographic distribution, citation patterns, keyword co-occurrence networks, thematic mapping, and international collaboration networks. Findings were complemented by a narrative synthesis of empirical studies to triangulate faculty development priorities across contexts.

**Results:**

Annual publication output increased eightfold, from 3 publications in 2005 to 24 in 2025, with a pronounced acceleration after 2018. Research output was concentrated in high-income countries, with the United States accounting for 717 citations compared to 69 (Australia) and 67 (Canada). Needs assessment was the most prevalent research theme (9% of all keywords), followed by staff development (6%) and curriculum (5%). Across diverse geographic and institutional settings, consistently reported faculty development priorities included teaching skills for large and small groups, assessment and feedback competencies, curriculum development, clinical supervision, and educational scholarship. The field relies predominantly on cross-sectional surveys and self-reported gap analyses; evaluation outcomes are largely restricted to Kirkpatrick Level 1 measures, with few studies examining behavioral change or institutional impact.

**Conclusion:**

Faculty development needs assessment research has grown substantially but remains geographically concentrated and methodologically limited. The consistency of priority needs across contexts highlights universal challenges in medical education, while subgroup variation underscores the importance of targeted, contextually responsive programming. Future research should adopt longitudinal evaluation designs, expand capacity in under-represented regions, and move beyond self-report to measure authentic educational impact.

## 1 Introduction

Faculty development has become a critical component of quality improvement in medical education, responding to the growing complexity of teaching, assessment, and educational research in health professions schools. As medical education evolves toward competency-based frameworks and workplace-based assessments, the need for systematic faculty development programs grounded in rigorous needs assessments has intensified. Competency-based medical education (CBME) requires faculty to develop competencies in workplace-based assessment, feedback, coaching, and competency-focused teaching. Within this context, needs assessment plays a critical role in identifying gaps in faculty preparedness and informing targeted faculty development initiatives that support effective CBME implementation (1–5). Understanding the landscape of faculty development research—its growth patterns, geographic distribution, thematic priorities, and methodological approaches—is essential for designing evidence-based programs that address authentic faculty needs.

This study presents a bibliometric analysis of faculty development needs assessment research in medical education, examining publication trends, author collaborations, citation patterns, conceptual structures, and thematic evolution over a 20-year period from 2005 to 2025. Bibliometric methods provide a systematic, quantitative approach to mapping the intellectual structure of a research field, revealing patterns that may not be apparent through traditional narrative reviews (6). By combining bibliometric findings with insights from empirical needs assessment studies, this analysis aims to provide a comprehensive overview of how the field has evolved, what priorities have emerged, and where gaps remain.

The analysis addresses several key questions: How has research output in faculty development needs assessment grown over time? Which countries, authors, and journals have been most influential? What are the dominant themes, and how have they evolved? What methodologies are most commonly employed for needs assessment? What priority needs do faculty consistently report across contexts? Understanding these patterns can inform future research directions and guide institutions in developing targeted, evidence-based faculty development initiatives.

The aim of this study was to map the global intellectual landscape of faculty development needs assessment research in medical education from 2005 to 2025, and to synthesize empirically identified faculty development priorities to inform evidence-based program design.

## 2 Methods

### 2.1 Search strategy and data collection

The bibliometric analysis was based on a systematic search of scholarly literature on faculty development needs assessment in medical education. A systematic bibliometric search was conducted on January 13,2026, across three major citation databases: Pub Med (NLM), Scopus (Elsevier), and Web of Science (WOS) Core Collection (Clarivate). These databases were selected for their comprehensive coverage of medical education and health professions research. The search strategy was developed in collaboration with a medical librarian and combined Medical Subject Heading (Me SH) and free -text terms related to faculty development, needs assessment, and medical education. The search was applied to titles, abstract, and author keywords (TITLE-ABS-KEY in Scopus and TS or [Topic] in WOS) and was adapted for each database as required. The search was restricted to English -Language Publications, including peer-reviewed journals articles published between January 2005 and December 2025.

Inclusion criteria were: (1) peer-reviewed publication in English; (2) explicit focus on faculty development needs in health profession education; and (3) publications between 2005 and 2025. Exclusion criteria were (1) editorials, letters, commentaries, and non-peer-reviewed publications; (2) studies not related to faculty development in medical education; and (3) duplicate records across database.

**FIGURE 1:**
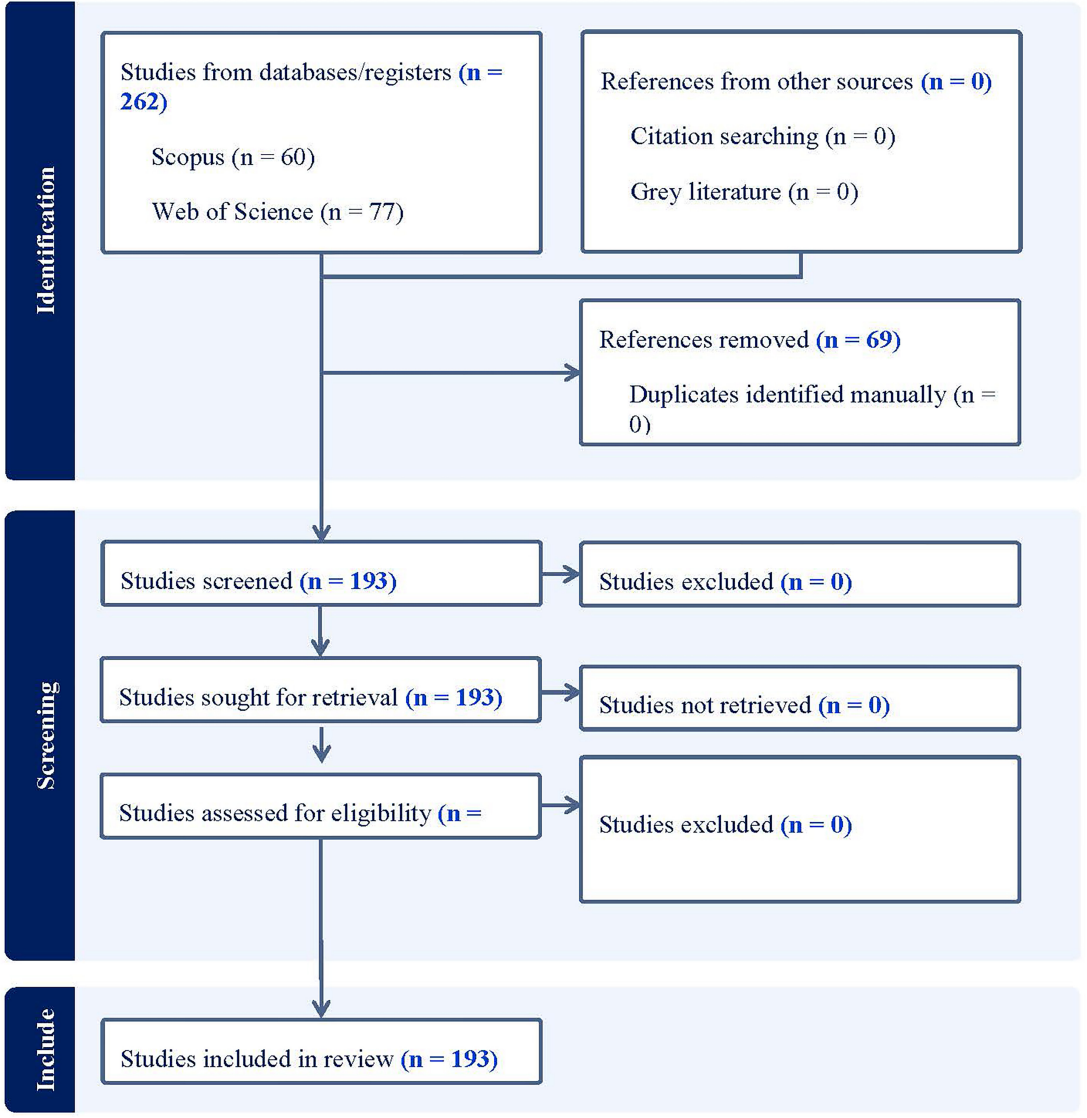
PRISMA flow diagram. Flow diagram illustrating the process of study identification, screening, eligibility assessment, and inclusion according to PRISMA guidelines.

The initial search identified 262 references. 69 duplicates records were automatically removed, leaving 193 unique references for analysis. The study selection process followed PRISMA 2020 guidelines, and a PRISMA flow diagram is provided as Figure 1. Bibliographic data extracted included publication year, author names, institutional affiliations, countries, journals, abstract, author keywords, citation counts, and Digital Object Identifiers (DOIs). The full, reproducible search strings and Boolean logic used for each database are detailed in Supplementary Material 1.

### 2.2 Bibliometric analysis approach

All bibliometric analyses were performed using Biblioshiny (version 5.1.0), the web-based interface of the Bibliometrix R package, under R version 4.4.1 (R Foundation for Statistical Computing, Vienna, Austria). The dataset was compiled from three databases—Web of Science, Scopus, and PubMed—and deduplicated using DOI and title/year matching. Where the same publication appeared across multiple databases, a single record was retained following the software’s default priority order (Web of Science, Scopus, then PubMed). Author names, institutional affiliations, and country data were manually reviewed and standardized to reduce cross-database inconsistencies; clearly distinct authors or institutions were kept separate. A custom synonym and exclusion list was applied to harmonize keyword terminology and remove irrelevant terms; the complete list is provided in Supplementary Material 2.

The research landscape was characterized across several complementary dimensions. Annual publication counts were tracked to assess scientific output over the study period, and geographic distribution was mapped by linking author affiliations to countries to compare publication volume and citation impact by region. Author productivity was measured by publication frequency, and journal contribution was evaluated using Bradford’s Law to identify core publication outlets. Citation analysis was used to determine the most influential publications and contributing countries.

Conceptual and collaborative structures were explored through network-based methods. Keyword co-occurrence networks were constructed to identify thematic clusters and their interrelationships, supplemented by a keyword tree map to illustrate term frequency. International collaboration patterns were assessed using country-level co-authorship networks. Thematic mapping followed the framework proposed by Cobo et al. (7), classifying themes into four quadrants—motor, niche, emerging/declining, and basic—based on centrality and density indices. Centrality reflects the degree of interaction between a theme and other themes within the research field, indicating its overall importance, whereas density reflects the internal development and cohesion of a theme. Motor themes (high centrality, high density) represent well-developed and influential topics; basic themes (high centrality, low density) are important but require further development; niche themes (low centrality, high density) are specialized and internally cohesive topics; and emerging or declining themes (low centrality, low density) represent either newly developing areas or topics receiving decreasing research attention. Trend-topic analysis was further applied to examine how research priorities evolved over time. For all network visualizations, networks were restricted to the top 50 nodes, edge weights were normalized using association strength, communities were detected via the Louvain clustering algorithm, and a repulsion force of 0.5 was applied. To contextualize and triangulate the bibliometric findings, empirical studies reporting faculty development needs assessments were narratively synthesized alongside the quantitative results, enabling identification of priority development needs across diverse institutional and geographic settings.

## 3 Results

### 3.1 Growth and global distribution of faculty development research

**FIGURE 2:**
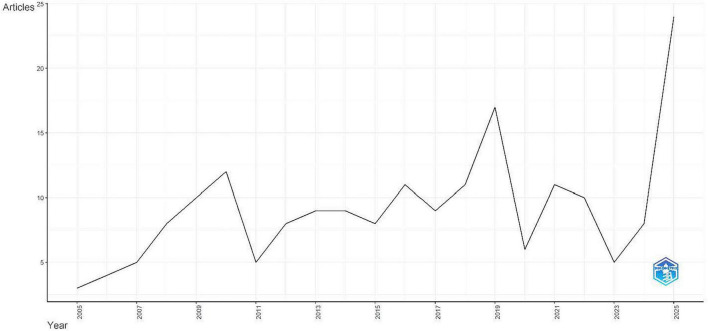
Annual scientific production. Line graph demonstrating the annual number of publications on faculty development needs assessment in medical education from 2005 to 2025, showing a marked increase after 2018.

Bibliometric analysis revealed a sustained and progressive expansion of faculty development research in medical education from 2005 to 2025. Annual scientific output increased markedly from 3 publications in 2005 to a peak of 24 in 2025 (Figure 2), representing an eightfold growth over the study period and reflecting deepening scholarly engagement with this domain. A pronounced acceleration in publication output was observed from 2018 onward, suggesting a paradigm shift in the prioritization of faculty development within contemporary medical education research agendas, potentially catalyzed by growing recognition of its centrality to educational quality and institutional capacity building.

**FIGURE 3:**
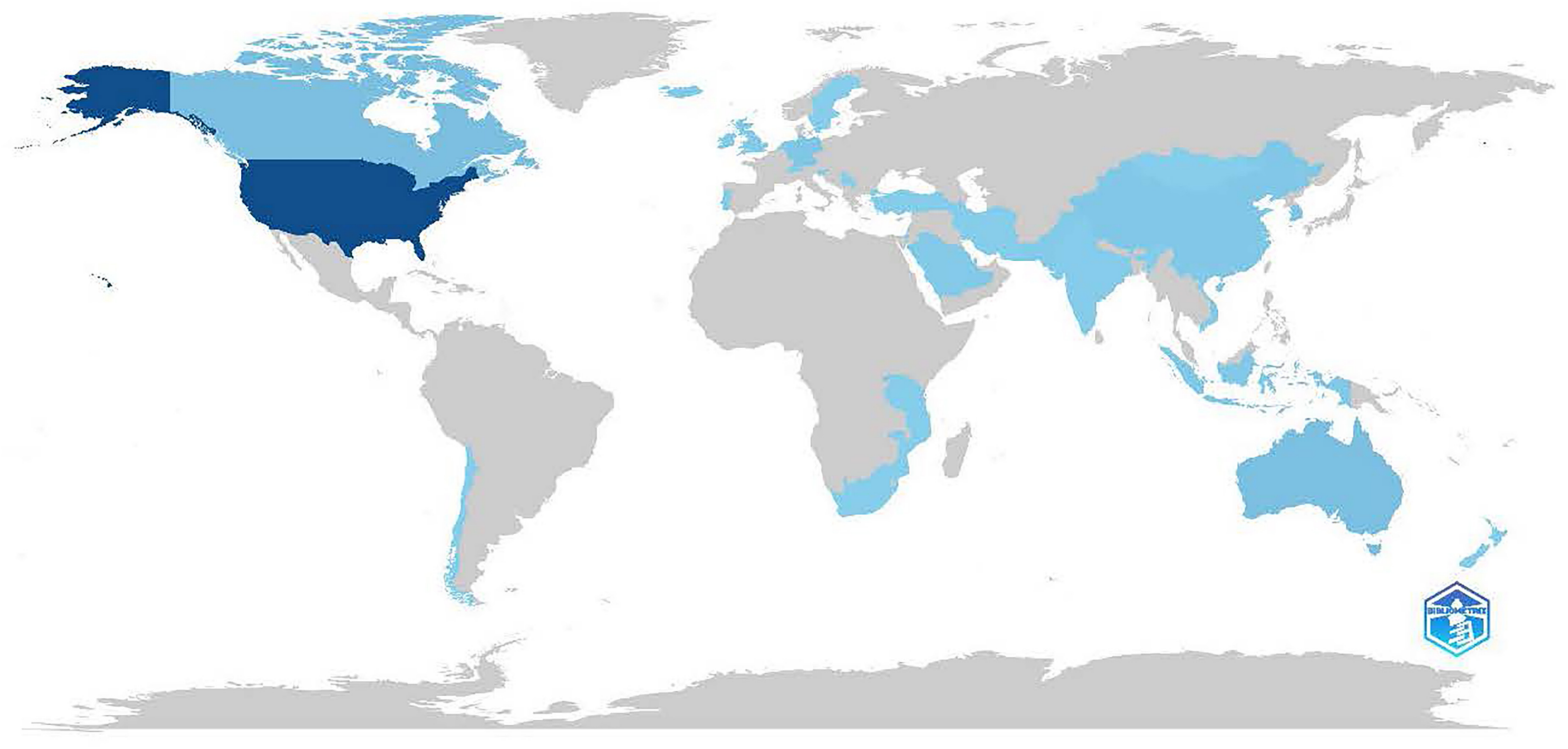
Country scientific production. Global distribution of publications in faculty development research showing countries contributing to the field, with darker shading representing higher publication output.

Geospatial analysis of publication distribution (Figure 3) revealed a notable concentration of research output in high-income countries. The United States emerged as the predominant contributor, followed by Canada, Australia, China, and several European nations. While nascent contributions from Asia and the Middle East signal an expanding global interest, the overall knowledge base remains predominantly Western-centric, underscoring persistent structural disparities in global research participation and capacity.

**FIGURE 4:**
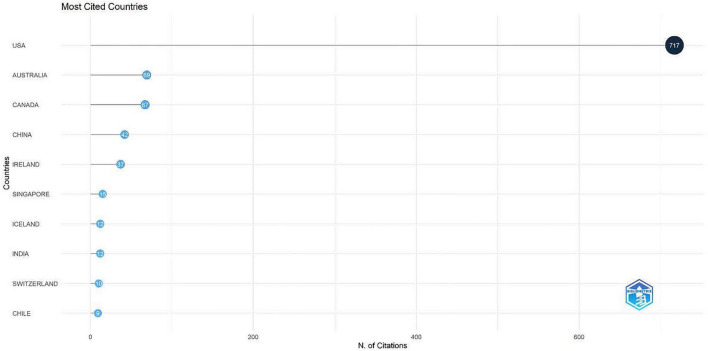
Most cited countries in faculty development research. Citation impact of countries contributing to faculty development literature, highlighting nations with the highest total citations.

Citation analysis further corroborated this geographic imbalance. The United States demonstrated substantially greater citation impact (717 citations) relative to other contributing nations, including Australia (69 citations) and Canada (67 citations) (Figure 4), indicating that scholarly influence remains disproportionately concentrated within a limited number of established research systems.

Despite growing international participation in terms of publication volume, the asymmetry between contributory breadth and citation reach reflects an enduring hierarchy in global knowledge production.

**FIGURE 5:**
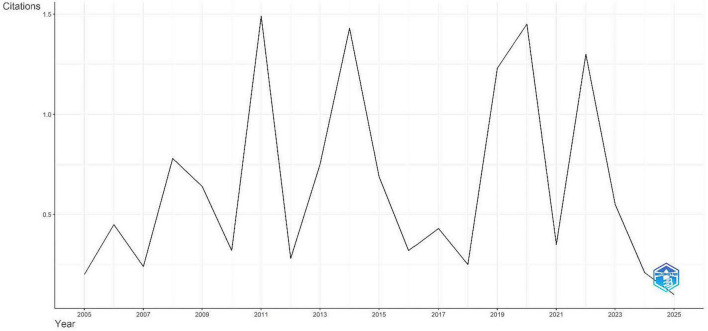
Average citations per article per year. Trend of average citation impact per year across the study period, illustrating fluctuations in scholarly influence over time.

Temporal analysis of citation impact revealed considerable variability in average citations per year, with distinct peaks identified around 2011, 2014, 2019, 2020 and 2022 (Figure 5). These inflection points likely correspond to the publication of landmark studies that exerted formative influence on subsequent research trajectories, shaping both conceptual frameworks and methodological approaches within the field. Collectively, these findings indicate that while faculty development research has achieved robust quantitative growth, its intellectual influence and citation reach remain geographically concentrated among a small cohort of leading research systems. Detailed country-level publication trends are provided in the Supplementary Figure 1.

### 3.2 Influential authors, sources, and collaboration networks

**FIGURE 6:**
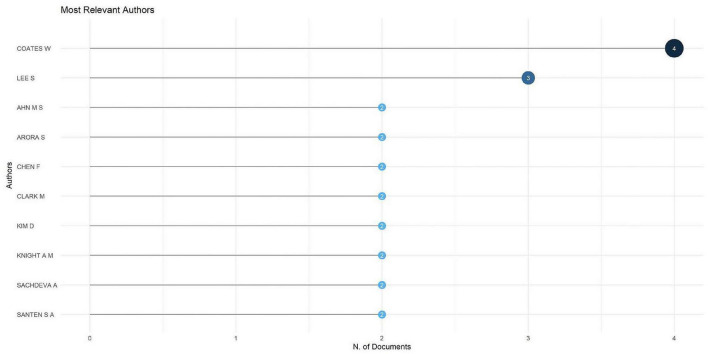
Most relevant authors. Leading authors ranked by number of publications in the dataset, highlighting the most productive contributors to faculty development scholarship.

Authorship analysis identified a relatively small but highly productive cohort of contributors driving scholarly output in this field. Wendy C. Coates was the most prolific author (*n* = 4 publications), followed by S. Lee (*n* = 3), with M. S. Ahn, S. Arora, and F. Chen each contributing two publications (Figure 6). Temporal patterns of author productivity (Supplementary Figure 2) suggest sustained longitudinal contributions from key individuals, indicative of the consolidation of specialized expertise and the emergence of an established scholarly community within the field.

**FIGURE 7:**
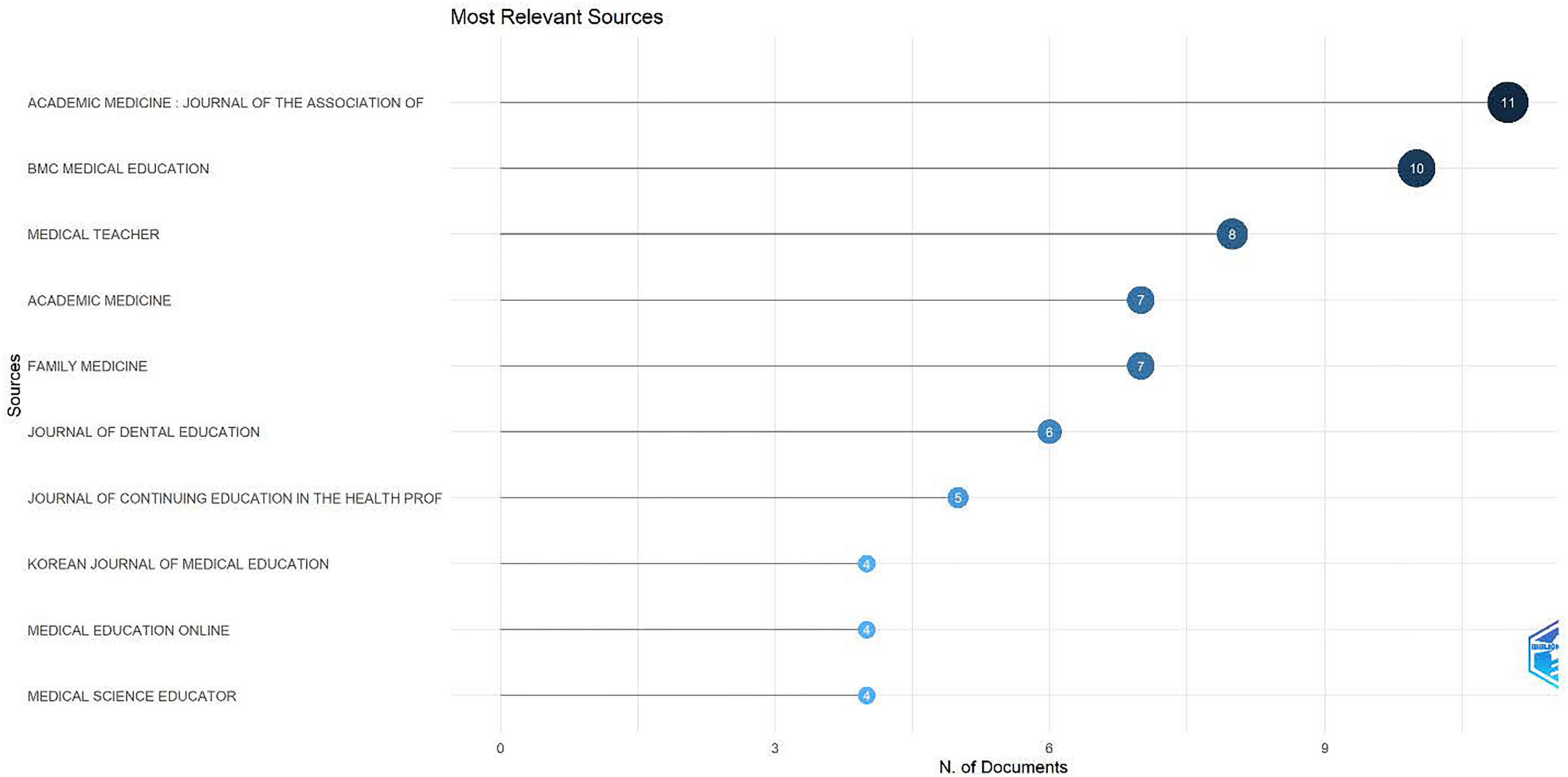
Most relevant sources. Journals with the highest number of publications in the field, indicating key outlets for faculty development research.

**FIGURE 8:**
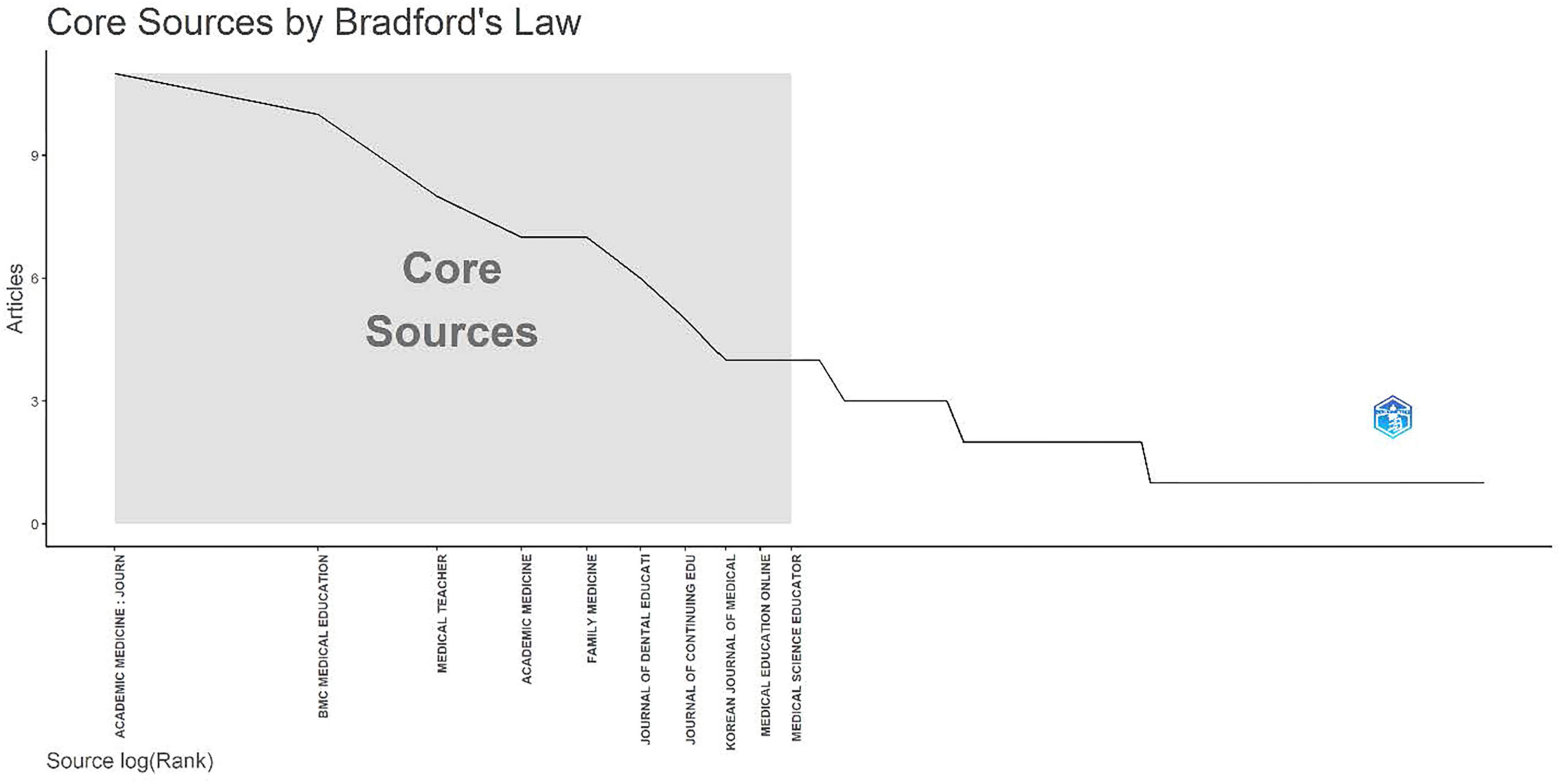
Core sources identified by Bradford’s Law. Distribution of journals according to Bradford’s Law illustrating the core set of sources responsible for most publications.

**FIGURE 9:**
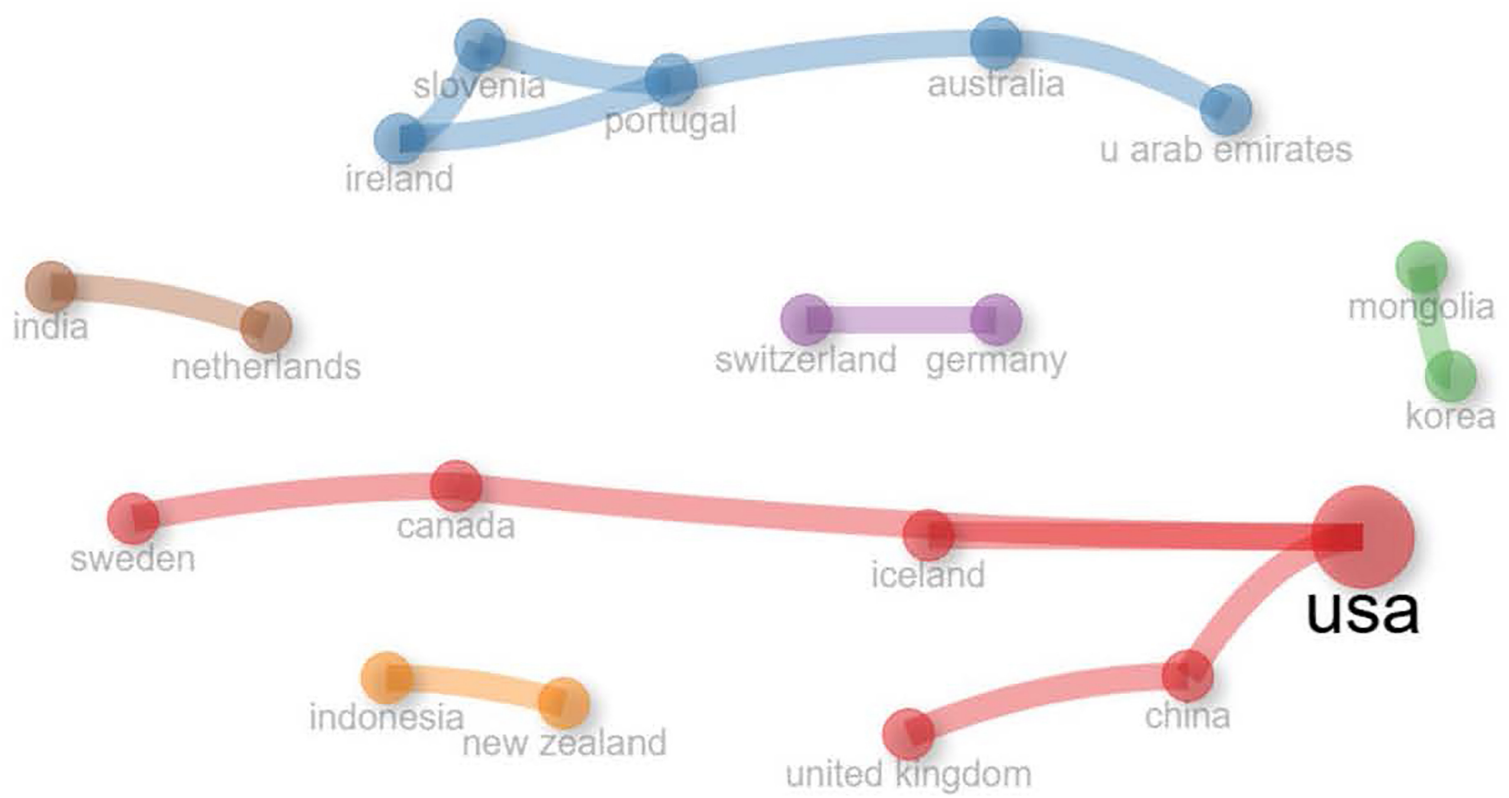
International collaboration network. Network visualization of collaboration among countries in faculty development research, where node size reflects publication activity and connecting lines represent collaborative relationships.

Source analysis demonstrated a concentration of publications within a core group of specialized medical education journals (Figure 7). Application of Bradford’s Law confirmed this distribution, delineating a core zone of journals responsible for a disproportionately large share of total output (Figure 8). The most prominent outlets were Academic Medicine (*n* = 11), BMC Medical Education (*n* = 10), and Medical Teacher (*n* = 8), affirming their central role as primary platforms for the dissemination of faculty development research. Supplementary analyses of additional source impact metrics, including journal H-index, are provided in Supplementary Figure 3, institutional productivity data are detailed in Supplementary Figure 4, and sources’ production over time is presented in Supplementary Figure 5. Collaboration network analysis (Figure 9) demonstrated a pattern of increasing international cooperation, with the United States functioning as a central hub connecting collaborators from Canada, the United Kingdom, Iceland, and China. Additional regional collaborative clusters were identified across Europe and the Asia–Pacific region, reflecting a progressively interconnected and geographically diverse research landscape. Nevertheless, collaboration networks remain notably skewed toward high-income countries, with limited engagement from low- and middle-income settings, a finding that warrants targeted strategies to foster more equitable and inclusive international partnerships.

### 3.3 Conceptual structure of the field

**FIGURE 10:**
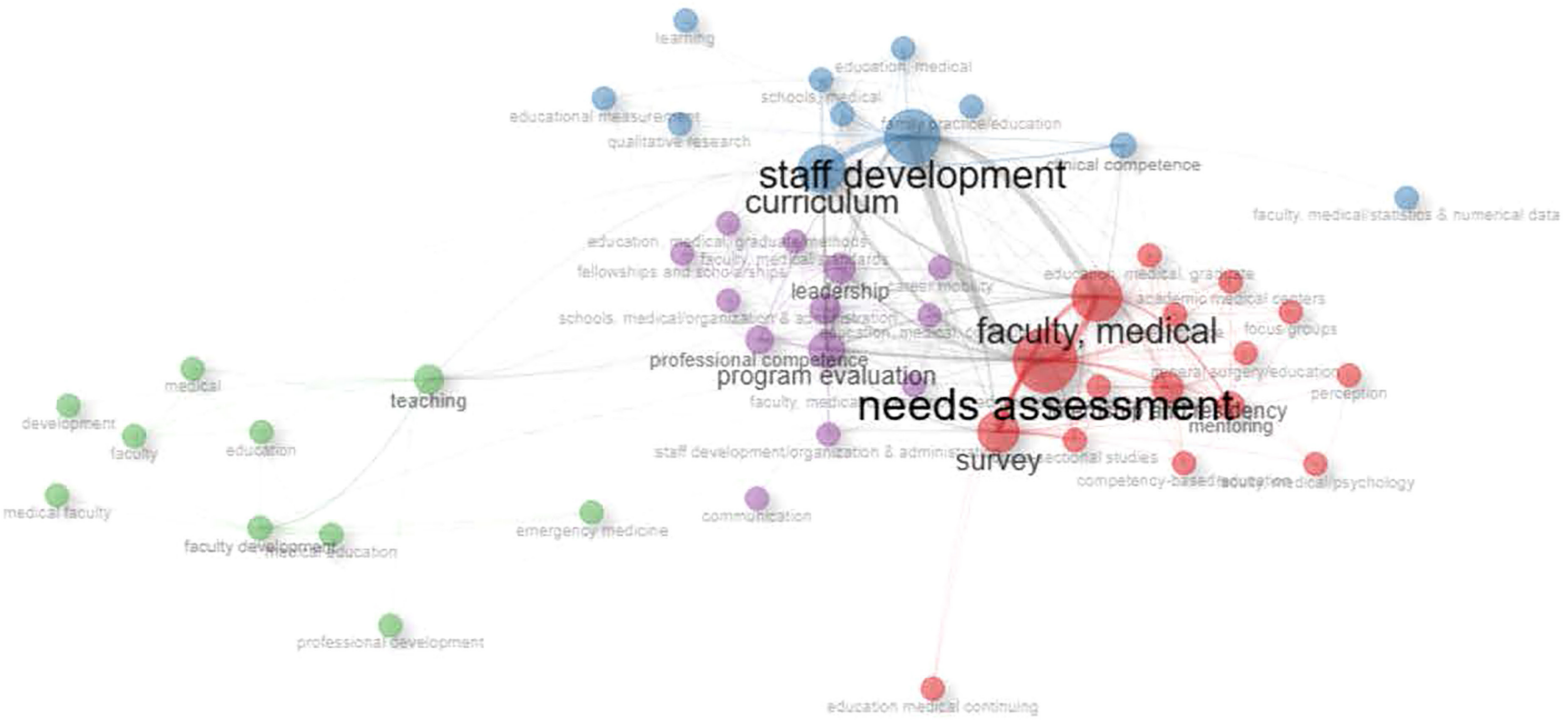
Keyword co-occurrence network. Network visualization of keyword co-occurrence illustrating conceptual relationships among major research topics in faculty development. Node size represents keyword frequency, while links indicate co-occurrence relationships between concepts.

Keyword co-occurrence analysis disclosed a well-defined and internally coherent conceptual structure underpinning faculty development research in medical education. As depicted in Figure 10, multiple interconnected thematic clusters were identified, with dominant conceptual nodes centered on faculty development, medical education, curriculum, mentoring, and needs assessment. The density of co-occurrence linkages among topics such as professional development, assessment strategies, and training interventions reflects strong conceptual integration, indicative of a cohesive and strategically focused research agenda oriented toward enhancing teaching competencies and educational quality. These patterns suggest that the field is primarily organized around three interrelated axes: advancing faculty pedagogical capabilities, refining assessment and developmental methodologies, and strengthening institutional support infrastructures. Supplementary analyses (Supplementary Figures 6, 7) corroborate these findings, illustrating the relative prominence and frequency distribution of key terms across the corpus.

**FIGURE 11:**
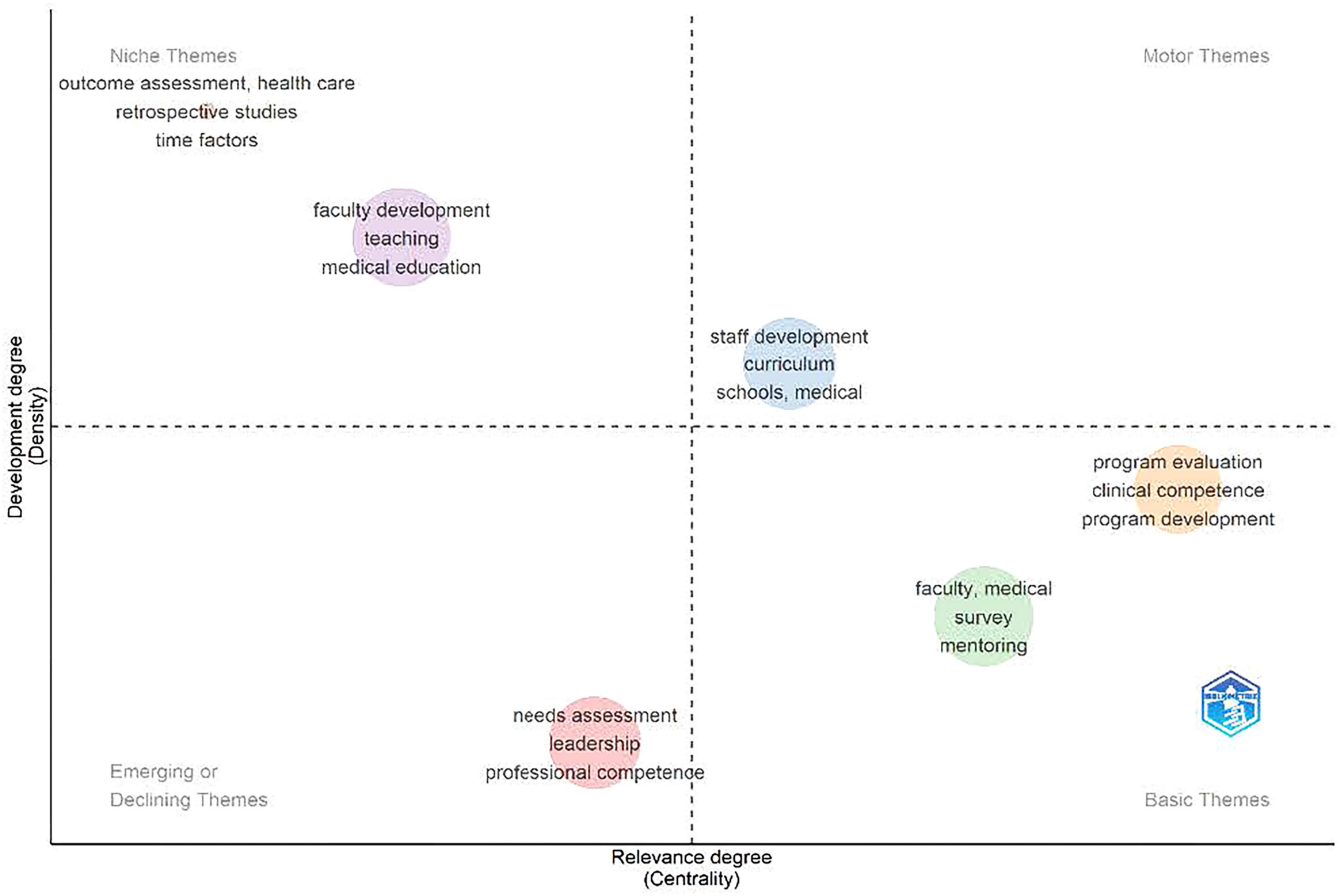
Thematic map. Thematic map generated from keyword co-occurrence analysis. The *x*-axis represents centrality (relevance of the theme within the research field), while the *y*-axis represents density (level of development of the theme). Bubble size reflects the frequency of keywords within each thematic cluster.

Thematic mapping provided a visual representation of the relative importance and development of research themes within the field. Motor themes characterized by high centrality and high density —denoting both scientific relevance and internal cohesion —included staff development, curriculum, and medical schools, identifying these as well-established, intellectually influential areas constituting the field’s conceptual core (Figure 11). By contrast, faculty development and teaching were positioned as niche themes, reflecting domains of specialized inquiry with comparatively limited cross-thematic connectivity. Needs assessment, leadership, and professional competence were classified as emerging or transitional themes, signaling growing scholarly interest and a trajectory toward greater centrality in future research. Foundational themes—including faculty (medical), survey methodology, and mentoring—were situated in the basic quadrant, affirming their enduring relevance as structural pillars of the discipline. These thematic patterns may help faculty development leaders identify established areas for continued investment, recognize emerging educational priorities, and guide the design of faculty development programs that are responsive to evolving institutional and learner needs.

**FIGURE 12:**
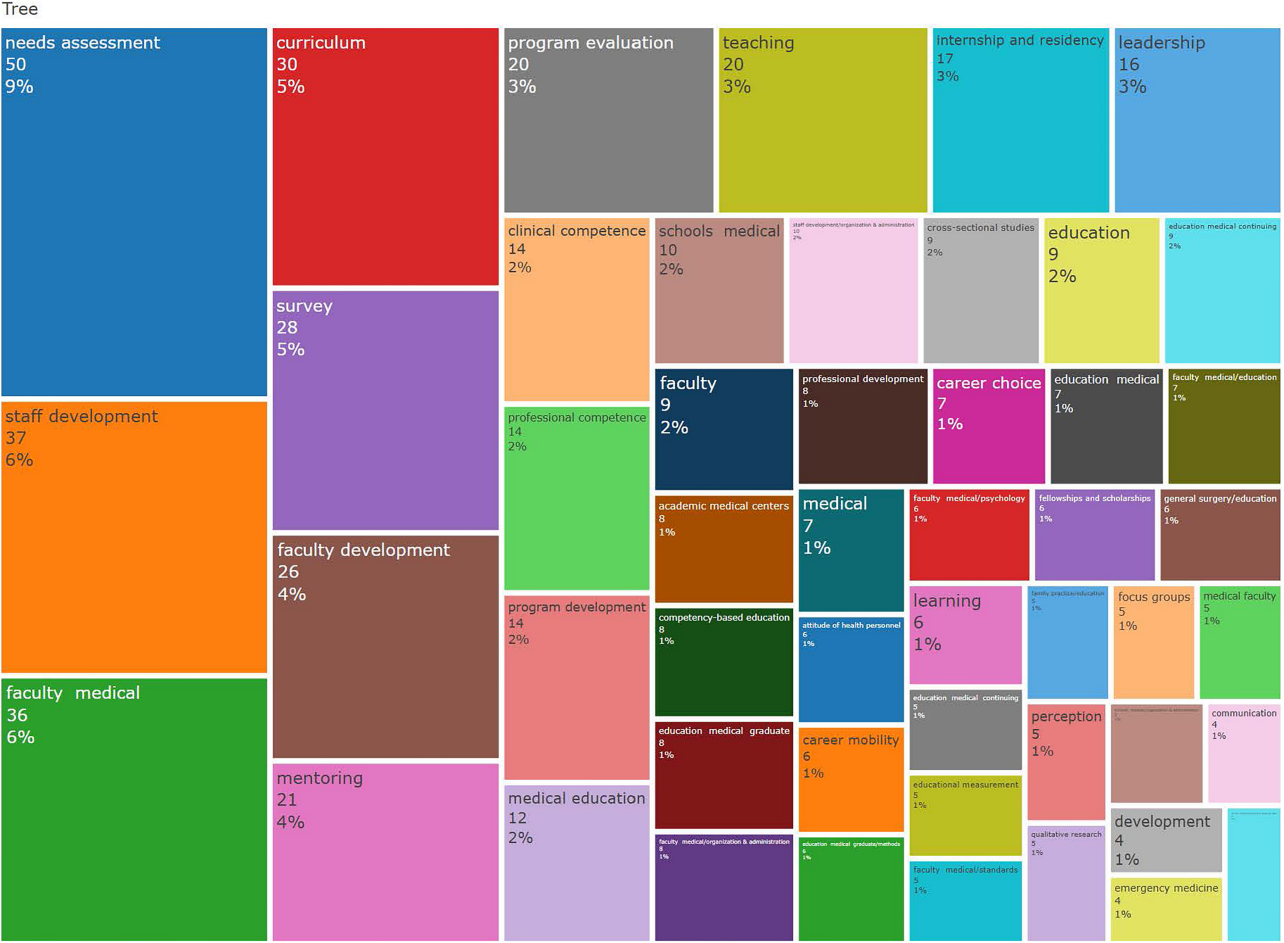
Treemap showing distribution of keywords. Treemap illustrating the relative frequency of keywords identified in the dataset. Larger rectangles represent keywords with higher occurrence in the literature.

Frequency analysis (Figure 12: Tree map) further highlighted the relative dominance of specific topics, with needs assessment emerging as the most prevalent subject (9%), followed by staff development (6%), faculty (medical) (6%), curriculum (5%), and survey methodology (5%). This distribution underscores the centrality of systematic evaluation of educational needs and the design of targeted faculty development interventions as defining preoccupations of the field.

Temporal trend analysis (Supplementary Figures 8, 9) revealed the sustained prominence of needs assessment, faculty development, and curriculum over the study period. More recent years have demonstrated increasing emphasis on professional development and methodological approaches such as cross-sectional study designs, reflecting both thematic broadening and a progressive diversification of research methodologies. This trajectory suggests that the field is evolving beyond descriptive inquiry toward more systematic, evidence-informed frameworks for faculty development practice and policy.

### 3.4 Needs assessment methodologies in literature

Needs assessments for faculty development in medical education predominantly employ cross-sectional surveys, structured gap analyses comparing importance versus performance, modified Delphi methods, and mixed-methods approaches including focus groups and interviews. Surveys typically quantify current versus desired competence, with gap-based rankings and summary scores such as the Borich formula used to prioritize topics for faculty development programs. Cross-sectional questionnaires, as employed in studies from Saudi Arabia, Pakistan, and South Korea, are efficient for broad sampling and ranking multiple domains but rely on self-reported data (7–11). Gap analysis methods, such as those used in Gulf medical schools, produce prioritized actionable gaps by combining perceived importance and performance ratings (12). The Borich needs assessment formula, applied at Dong-A University, provides a quantitative prioritization of specific competencies based on perceived importance and ability scores (11). Modified Delphi techniques have been used to build specialty-relevant instruments and achieve content validity, as demonstrated in pediatric critical care faculty development (12). Mixed-methods approaches, including focus group discussions, add depth to survey findings and aid in indicator generation and contextualization (13). Common assessment instruments and tools cited as targets of faculty development include workplace-based assessment forms, Objective Structured Assessment of Technical Skills (OSATS), Zwisch scales, and structured clinical examinations. Studies note both the need to train faculty in these tools and low familiarity among some educators (14). Surveys are often delivered online and stratified by rank, discipline, or institution to detect subgroup differences (15–16).

### 3.5 Priority faculty development needs

Across diverse geographic and institutional contexts, common high priority needs consistently emerge from needs assessment studies. Teaching skills for large and small groups are among the most frequently cited priorities, with studies from Saudi Arabia, Bangladesh, and Canada reporting strong faculty interest in improving instructional methods (8, 9, 10). Assessment and feedback competencies represent another critical area of need, with faculty reporting gaps in providing high-quality verbal and written feedback and in using specific assessment tools, particularly for non-technical skills (11, 14).

Curriculum development and course evaluation are consistently identified as priority areas, with faculty expressing needs in developing learning resources, planning curricula, and building assessment frameworks such as Objective Structured Clinical Examinations (9, 10, 17). Clinical supervision and workplace-based assessment have gained prominence with the implementation of competency-based medical education frameworks, and Canadian emergency medicine faculty have identified feedback delivery and completion of workplace-based assessments as top priorities (18). Educational research and scholarship represent another major need area, with faculty seeking training in research methods, statistical analysis, and pathways to advanced degrees in health professions education (17, 18, 19). Studies also report needs in leadership development, non-technical skills, and e-learning platforms, reflecting the evolving demands of medical education (8, 9).

Variation in needs by subgroups has been documented, with junior faculty and clinician-educators often reporting greater needs than senior faculty, and some gender and discipline differences observed in priorities (15, 16, 20). Female academic emergency physicians, for example, expressed greater overall faculty development needs in leadership compared to males (18). Assistant professors showed higher educational needs across most competencies compared to other faculty groups (10). Faculty members demonstrate strong willingness to participate in development programs when topics are relevant and delivery is accessible. Studies from Pakistan reported that nearly 95% of teachers are willing to attend faculty development workshops (9). Preferred learning modalities include brief colleague discussions, short videos (10 min), regular conference series, and hands-on workshops, highlighting a need for efficient, workplace-friendly formats that can be integrated into regular professional activities (8, 12, 21).

## 4 Discussion

### 4.1 Growth and maturation of the field

The eightfold increase in annual publications from 2005 to 2025 demonstrates that faculty development needs assessment has matured into a recognized research domain within medical education. The acceleration after 2018 coincides with widespread adoption of competency-based medical education frameworks, which place new demands on faculty for direct observation, formative feedback, and workplace-based assessment. This growth pattern suggests that faculty development research is responsive to broader educational reforms and policy shifts in health professions education.

The variable citation peaks around 2011, 2014, 2019, 2020 and 2022 indicate periods when particularly influential work shaped the field’s trajectory. These peaks may correspond to landmark publications, systematic reviews, or methodological innovations that catalyzed subsequent research. The sustained growth in publication volume, combined with periodic high-impact contributions, suggests a field that is both expanding and consolidating its knowledge base.

### 4.2 Geographic concentration and global equity

The concentration of research output and citation impact in high-income countries, particularly the United States, raises important questions about global equity in faculty development research. While the United States’ leadership in publication volume and citations (717 citations) reflects substantial investment in medical education research infrastructure, the significant gap between the U.S. and other contributors such as Australia (69 citations) and Canada (67 citations) suggests that research capacity remains unevenly distributed. The emergence of contributions from Asia and the Middle East, including studies from Pakistan, Saudi Arabia, South Korea, and China, indicates growing global engagement with faculty development needs assessment. However, the continued dominance of Western research paradigms and publication outlets may not fully capture the diverse contexts, priorities, and challenges faced by medical educators in low- and middle-income countries. The international collaboration networks, while valuable, remain centered on high-income countries, suggesting that South-South collaboration and knowledge exchange may be underdeveloped. Future research should prioritize building research capacity in underrepresented regions, supporting local publication outlets, and ensuring that faculty development frameworks are culturally responsive and contextually appropriate. Interpretation of the geographic distribution of publications should also consider the potential influence of language bias, as this study was restricted to English-language publications. Consequently, scholarship from settings where local-language publication remains common may be underrepresented in the present analysis.

### 4.3 Dominant themes and research priorities

The prominence of needs assessment as the most dominant research theme (9% of all topics) reflects a field that recognizes the importance of evidence-based program design. The strong linkages between needs assessment, professional development, curriculum, and training indicate an integrated research agenda that views faculty development as a systematic, ongoing process rather than isolated interventions. This pattern can be further understood through established theories of professional learning. Communities of Practice highlights how professional learning occurs through participation in educational communities and collaborative knowledge sharing, while Transformative Learning Theory emphasizes critical reflection as a mechanism for reshaping professional perspectives and practices (22–26). Social Cognitive Theory further suggests that self-efficacy, observational learning, and social interactions influence behavior change and facilitate the transfer of learning into educational and clinical practice (26). The identification of needs assessment, leadership, and professional competence as emerging or transitional topics suggests that the field is evolving toward more sophisticated conceptualizations of faculty development. The sustained prominence of traditional themes such as teaching, curriculum, and mentoring alongside emerging priorities such as workplace-based assessment and competency-based education indicates that faculty development must address both foundational competencies and new demands. The continued prominence of mentoring highlights its importance as a key mechanism within faculty development. Mentoring and coaching relationships contribute to professional identity formation, career progression, and sustained professional growth through guidance, feedback, reflective practice, and role modeling. As longitudinal developmental processes, these relationships complement formal faculty development initiatives by supporting continuous learning and adaptation across academic career stages (27–32). The consistency of priority needs across diverse contexts—teaching skills, assessment and feedback, curriculum development, clinical supervision, and educational scholarship—suggests that these represent universal challenges in medical education. However, the variation by subgroups (rank, gender, discipline) indicates that one-size-fits-all approaches are insufficient. Effective faculty development programs must balance common core competencies with targeted interventions for specific faculty populations.

### 4.4 Methodological patterns and limitations

The predominance of cross-sectional surveys and self-reported needs assessments reflects pragmatic constraints in faculty development research, including limited resources, time pressures, and the challenge of measuring complex educational outcomes. While these methods are efficient for identifying perceived needs and prioritizing topics, they have important limitations. Self-report data may be influenced by social desirability bias, limited self-awareness, or discrepancies between perceived and actual competence.

The systematic review finding that 23 studies addressed only Kirkpatrick Level 1 (reaction) while only 4 addressed Level 4 (results) highlights a critical gap in the evidence base (12). Most studies report short-term outcomes such as improved confidence and perceived readiness, with limited objective data on behavior change, educational impact, or patient outcomes. This pattern limits the field’s ability to demonstrate the value of faculty development investments and to identify which program features are most effective.

Previous studies have highlighted the importance of evaluating higher-level outcomes, particularly sustained behavior change and organizational impact, which remain underrepresented in faculty development research. Longitudinal study designs, direct observation of teaching practices, teaching portfolios, multi-source feedback, and measures of scholarly or educational outputs may provide more robust evidence of behavioral change beyond self-reported outcomes (25, 33–36). At the organizational level, evaluation frameworks incorporating institutional metrics, communities of practice, and mixed-methods approaches may help capture broader effects on educational culture, practice, and performance (34). The use of gap analysis methods and the Borich formula represents methodological sophistication in prioritizing needs, but these approaches still rely on self-assessment. The modified Delphi technique and mixed-methods approaches that incorporate focus groups and interviews add valuable depth and context but remain underutilized. Future research should prioritize longitudinal designs, objective performance measures, and multi-level evaluation frameworks that assess impact on teaching behavior, learner outcomes, and institutional culture. The low familiarity among faculty with assessment tools such as OSATS and Zwisch, as documented in surgical education, underscores the gap between educational innovation and faculty preparedness (14). This finding suggests that faculty development must not only address generic teaching skills but also provide specific training in contemporary assessment methods and educational technologies.

### 4.5 Limitations of the present study

Several limitations of this bibliometric analysis warrant acknowledgement. First, restriction of the search to Scopus and Web of Science may have excluded relevant publications indexed only in regional or discipline-specific databases such as ERIC, EMBASE, or CINAHL. Second, analysis was confined to English-language publications, which may underrepresent scholarship produced in other languages, particularly from non-Western contexts. Third, citation counts inherently favors older publications and may not accurately reflect the current influence of more recently published work. Fourth, keyword co-occurrence analysis is dependent on author-assigned keywords, which introduces variability and may not fully capture the conceptual content of individual articles. Fifth, this study does not evaluate the quality of included publications, which limits interpretation of citation-based influence metrics. These limitations should be considered when interpreting and generalizing the findings.

### 4.6 Implementation implications

The literature provides important guidance for implementing effective faculty development programs. The strong faculty willingness to participate when topics are relevant and delivery is accessible (95% in some studies) indicates that lack of interest is not the primary barrier (9). Instead, barriers relate to time constraints, competing priorities, and program design. Preferred learning modalities—brief colleague discussions, short videos, regular conference series, and hands-on workshops—emphasize the need for efficient, workplace-integrated formats (13, 21). The importance of supervisor endorsement and institutional support suggests that faculty development must be embedded in organizational culture and aligned with promotion criteria and performance expectations. The recommendation for longitudinal programs rather than one-time workshops reflects evidence that sustained engagement is necessary for meaningful skill development and behavior change (37, 38). The emphasis on needs-based, contextualized programming suggests that generic faculty development is less effective than targeted interventions tailored to specific faculty populations, disciplines, or institutional contexts. The identification of competency-based medical education implementation as a driver of faculty development needs suggests that major curricular reforms create windows of opportunity for faculty development. Institutions should anticipate faculty development needs when planning educational innovations and allocating resources accordingly.

## 5 Conclusion

This bibliometric analysis reveals a growing and maturing field of faculty development needs assessment research, characterized by increasing publication volume, geographic concentration, and consistent thematic priorities. Teaching skills, assessment and feedback, curriculum development, clinical supervision, and educational scholarship emerged as common priority needs across diverse contexts, although subgroup variation underscores the importance of targeted interventions.

Methodologically, the field continues to rely heavily on cross-sectional surveys and self-reported needs, with limited longitudinal evaluation and objective outcome measurement. Research output and collaboration networks remain concentrated in high-income countries, raising important questions regarding global equity and the generalizability of findings.

Collectively, these findings indicate that faculty development needs assessment has become an established area of scholarship and reinforce the importance of needs-based, contextually tailored, and longitudinal faculty development programs. Future research should prioritize rigorous evaluation of behavior change and organizational outcomes, strengthen research capacity in underrepresented regions, and investigate how needs assessment findings can be translated into sustainable and effective faculty development initiatives. The consistent willingness of faculty to engage in relevant and accessible development opportunities provides an encouraging foundation for these efforts. Grounding faculty development in rigorous needs assessment, evidence-based program design, and systematic outcome evaluation offers a clear path toward strengthening teaching capacity and educational scholarship in health professions education.

## Data Availability

The original contributions presented in this study are included in this article/Supplementary material, further inquiries can be directed to the corresponding author.

## References

[1] Cooper D Holmboe E. Competency-based medical education at the front lines of patient care. *N Engl J Med.* (2025) 393:1549–50. 10.1056/NEJMc2512926 40700689

[2] Lee G Chiu A. Assessment and feedback methods in competency-based medical education. *Ann Allergy Asthma Immunol.* (2022) 128:256–62. 10.1016/j.anai.2021.12.010 34929390

[3] Fraser A Stodel E Jee R Dubois D Chaput A. Preparing anesthesiology faculty for competency-based medical education. *Can J Anaesth.* (2016) 63:1364–73. 10.1007/s12630-016-0739-2 27646528

[4] Mahan J Kaczmarczyk J Miller Juve A Cymet T Shah B Daniel R et al. Clinician educator milestones: assessing and improving educators’. *Skills. Acad Med.* (2024) 99:592–8. 10.1097/ACM.0000000000005684 38442199 PMC11520343

[5] Robertson M Cofrancesco J Levine R. Transforming individuals and institutions through competency-based faculty development. *Front Med.* (2025) 12:1621376. 10.3389/fmed.2025.1621376 40662075 PMC12256433

[6] Donthu N Kumar S Mukherjee D Pandey N Lim W. How to conduct a bibliometric analysis: an overview and guidelines. *J Bus Res.* (2021) 133:285–96. 10.1016/j.jbusres.2021.04.070

[7] Cobo M López-Herrera A Herrera-Viedma E Herrera F. An approach for detecting, quantifying, and visualizing the evolution of a research field: a practical application to the fuzzy sets theory field. *J Informetr.* (2011) 5:146–66. 10.1016/j.joi.2010.10.002

[8] Algahtani H Shirah B Subahi A Aldarmahi A Algahtani R. Effectiveness and needs assessment of faculty development programme for medical education: experience from Saudi Arabia. *Sultan Qaboos Univ Med J.* (2020) 20:e83–9. 10.18295/squmj.2020.20.01.012 32190374 PMC7065691

[9] Shah N Tabassum A Shah N. A needs assessment for faculty development at two medical colleges of Dow university of health sciences, Karachi. *Pak J Med Sci.* (2018) 34:1386–91. 10.12669/pjms.346.16302 30559790 PMC6290210

[10] Zehra T Saeed S Ali R Sultan A Hussain A. Needs assessment for faculty development in health professions education at a medical university in Karachi. *Pakistan. J Pak Med Assoc.* (2023) 73:147–9. 10.47391/JPMA.5229 36842026

[11] Si J. Needs assessment for developing teaching competencies of medical educators. *Korean J Med Educ.* (2015) 27:177–86. 10.3946/kjme.2015.27.3.177 26330068 PMC8813424

[12] Kumar A Atwa H Shehata M Al Ansari A Deifalla A. Faculty development programmes in medical education in the Eastern Mediterranean Region: a systematic review. *East Mediterr Health J.* (2022) 28:362–80. 10.26719/emhj.22.014 35670441

[13] Bone JN Szmuilowicz E Bembea MM Czaja A Storgion S Turner D et al. Expanding faculty development of teaching skills: a national needs assessment of pediatric critical care medicine faculty. *Pediatr Crit Care Med.* (2020) 21: e00283-90. 10.1097/PCC.0000000000002265 32150125

[14] Steinemann S Korndorffer J Dent D Rucinski J Newman R Blair P et al. Defining the need for faculty development in assessment. *Am J Surg.* (2021) 222:679–84. 10.1016/j.amjsurg.2021.06.010 34226039

[15] Schönwetter D Hamilton J Sawatzky J. Exploring professional development needs of educators in the health sciences professions. *J Dent Educ.* (2015) 79:113–23. 10.1002/j.0022-0337.2015.79.2.tb05865.x 25640615

[16] Scarbecz M Russell C Shreve R Robinson M Scheid C. Faculty development to improve teaching at a health sciences center: a needs assessment. *J Dent Educ.* (2011) 75:145–59. 10.1002/j.0022-0337.2011.75.2.tb05032.x 21293037

[17] Barzegar M Miri H Abedini S Kamali F Boushehri EA. comprehensive “real need” assessment, a step toward improving the quality of faculty development programs: a survey-based study in Hormozgan University of Medical Sciences. *Health Sci Rep.* (2024) 7:e2097. 10.1002/hsr2.2097 38736474 PMC11082091

[18] Brown G Lang E Patel K McRae A Chung B Yoon P et al. A national faculty development needs assessment in emergency medicine. *CJEM.* (2016) 18:161–82. 10.1017/cem.2015.77 26350557

[19] Manzoor I Zeeshan S Iqbal A Sarfraz F. Needs assessment for establishing faculty development program in a private medical college at Lahore. *J Ayub Med Coll Abbottabad.* (2018) 30:539–43. 30632332

[20] Adkoli B Al-Umran K Al-Sheikh M Deepak K. Innovative method of needs assessment for faculty development programs in a Gulf medical school. *Educ Health.* (2010) 23:389. 21290357

[21] Paige J Khamis N Cooper J. Learning how to “teach one”: a needs assessment of the state of faculty development within the consortium of the American college of surgeons accredited education institutes. *Surgery.* (2017) 162:1140–7. 10.1016/j.surg.2017.06.016 28811044

[22] Findyartini A Nirula L Mandache M Caramori U Cilliers F Cantillon P et al. From criteria to impact: the ASPIRE framework as a roadmap for faculty development excellence in health professions education. *Med Teach.* (2025) 47:1948–60. 10.1080/0142159X.2025.2507150 40411806

[23] Kitto S Fantaye A Ghidinelli M Andenmatten K Thorley Wiedler J de Boer K. Barriers and facilitators to the cultivation of communities of practice for faculty development in medical education: a scoping review. *Med Teach.* (2025) 47:1654–68. 10.1080/0142159X.2025.2495628 40271991

[24] Nair B Bleasel J Mwangi F Malau-Aduli B. Reimagining faculty development: a paradigm shift from content to transformative learning processes. *Med Teach.* (2025) 47:976–84. 10.1080/0142159X.2024.2390035 39154226

[25] Kohan M Changiz T Yamani N. A systematic review of faculty development programs based on the Harden teacher’s role framework model. *BMC Med Educ.* (2023) 23:910. 10.1186/s12909-023-04863-4 38037063 PMC10690997

[26] Mann K. Theoretical perspectives in medical education: past experience and future possibilities. *Med Educ.* (2011) 45:60–8. 10.1111/j.1365-2923.2010.03757.x 21155869

[27] Ramani S Kusurkar R Lyon-Maris J Pyörälä E Rogers G Samarasekera D et al. Mentorship in health professions education - an AMEE guide for mentors and mentees: AMEE Guide No. 167. *Med Teach.* (2024) 46:999–1011. 10.1080/0142159X.2023.2273217 37909275

[28] Vaa Stelling B Andersen C Suarez D Nordhues H Hafferty F Beckman T et al. Fitting in while standing out: professional identity formation, imposter syndrome, and burnout in early-career faculty physicians. *Acad Med.* (2023) 98:514–20. 10.1097/ACM.0000000000005049 36512808

[29] Wald H. Professional identity (trans)formation in medical education: reflection, relationship, resilience. *Acad Med.* (2015) 90:701–6. 10.1097/ACM.0000000000000731 25881651

[30] Love L Simonsen K Beck Dallaghan G Bowler C. Navigating faculty coaching: a framework of key considerations. *Acad Med.* (2026) 101:496–505. 10.1093/acamed/wvag015 41581128

[31] Sachdeva A. Preceptoring, proctoring, mentoring, and coaching in surgery. *J Surg Oncol.* (2021) 124:711–21. 10.1002/jso.26585 34212384

[32] Misky G Sharpe B Weaver A Niranjan-Azadi A Gupta A Rennke S et al. Faculty development in academic hospital medicine: a scoping review. *J Gen Intern Med.* (2023) 38:1955–61. 10.1007/s11606-023-08089-4 36877213 PMC10271943

[33] Allen L Hay M Palermo C. Evaluation in health professions education-Is measuring outcomes enough? *Med Educ.* (2022) 56:127–36. 10.1111/medu.14654 34463357

[34] Alexandraki I Rosasco R Mooradian A. An evaluation of faculty development programs for clinician-educators: a scoping review. *Acad Med.* (2021) 96:599–606. 10.1097/ACM.0000000000003813 33116061

[35] Alexandraki I Rosasco R Mooradian A. An evaluation of faculty development programs for clinician-educators: a scoping review. *Acad Med.* (2021) 96:599–606. 10.1097/ACM.0000000000003813 33116061

[36] Onyura B Ng S Baker L Lieff S Millar B Mori B. A mandala of faculty development: using theory-based evaluation to explore contexts, mechanisms and outcomes. *Adv Health Sci Educ Theory Pract.* (2017) 22:165–86. 10.1007/s10459-016-9690-9 27295217

[37] Steinert Y Mann K Centeno A Dolmans D Spencer J Gelula M et al. A systematic review of faculty development initiatives designed to improve teaching effectiveness in medical education: BEME Guide No. 8. *Med Teach.* (2006) 28:497–526. 10.1080/01421590600902976 17074699

[38] Steinert Y Mann K Anderson B Barnett B Centeno A Naismith L et al. A systematic review of faculty development initiatives designed to enhance teaching effectiveness: a 10-year update: BEME Guide No. 40. *Med Teach.* (2016) 38:769–86. 10.1080/0142159X.2016.1181851 27420193

