# Supplementary material for: Mapping faculty development needs in medical education: a bibliometric analysis
Source: Front Med (Lausanne). 2026 Jul 1;13:1858624. doi: 10.3389/fmed.2026.1858624 (PMC13370283; doi:10.3389/fmed.2026.1858624)
Supplement: Supplementary file 1 [file Data_Sheet_1.PDF]

Supplementary Material 1

Table S1: Search strategy and database coverage for the bibliometric analysis

Detailed search queries used in Web of Science, Scopus, and PubMed, including database coverage, applied filters, and number of records retrieved from each source. A total of 262 records were identified across all databases, with 193 unique records remaining after deduplication.

Source and coverage	Search string	Results	notes
**PubMed (NLM)**<br>**Coverage: From database inception-2026-01-15**	(((((((((((((((Medical Faculty[MeSH Terms]) OR ("Medical Faculty"[Title/Abstract])) OR ("Medical Faculties"[Title/Abstract])) OR ("Academic Medical Faculty"[Title/Abstract])) OR ("Clinical Faculty"[Title/Abstract])) OR ("Medical Educator*"[Title/Abstract])) OR ("Medical Teacher*"[Title/Abstract])) OR ("Medical Instructor*"[Title/Abstract])) OR ("Health Professions Faculty"[Title/Abstract])) OR ("Academic Physician*"[Title/Abstract])) OR ("Physician Educator*"[Title/Abstract])) OR ("Clinical Educator*"[Title/Abstract])) OR ("Medical School Faculty"[Title/Abstract])) OR ("Academic Medicine Faculty"[Title/Abstract])) OR ("Basic Science Faculty"[Title/Abstract])) OR ("Preclinical Faculty"[Title/Abstract])) OR ("Medical Education Faculty"[Title/Abstract])) AND (((((((((((((((((((((Needs Assessment[MeSH Terms]) OR (Educational Needs Assessment[MeSH Terms])) OR ("Educational Need*"[Title/Abstract])) OR ("Training Need*"[Title/Abstract])) OR ("Professional Need*"[Title/Abstract])) OR ("Development Need*"[Title/Abstract])) OR ("Learning Need*"[Title/Abstract])) OR ("Faculty Need*"[Title/Abstract])) OR ("Gap Analysis"[Title/Abstract])) OR ("Training Gap*"[Title/Abstract])) OR ("Educational Gap*"[Title/Abstract])) OR ("Competency Gap*"[Title/Abstract])) OR ("Skill*[Title/Abstract])) OR (Gap*"[Title/Abstract])) OR ("Needs Identification"[Title/Abstract])) OR ("Needs Prioritization"[Title/Abstract])) OR ("Priority Setting"[Title/Abstract])) OR ("Needs Analysis"[Title/Abstract])) OR ("Training Requirement*"[Title/Abstract])) OR ("Educational Requirement*"[Title/Abstract])) OR ("Competency Assessment"[Title/Abstract])) OR ("Skills Assessment"[Title/Abstract])) OR ("Professional Development Need*"[Title/Abstract]))) AND ((((((((((((Faculty Development[MeSH Terms]) OR (Staff Development[MeSH Terms])) OR ("Professional Development"[Title/Abstract])) OR ("Human Resources Development"[Title/Abstract])) OR ("Academic Development"[Title/Abstract])) OR ("Capacity Building"[Title/Abstract])) OR ("Faculty Training"[Title/Abstract])) OR ("Academic Staff Development"[Title/Abstract])) OR ("Continuing	125 results	All search terms are searched in the field: [Title/Abstract] and in MeSH (when available). filters or English language, articles, Years 2005 - 2025

	Medical Education"[Title/Abstract])) OR ("Professional Growth"[Title/Abstract])) OR ("Continuing Professional Development"[Title/Abstract])) OR ("Educational Development"[Title/Abstract])) OR ("Teaching Development"[Title/Abstract])) OR ("Academic Capacity Building"[Title/Abstract])) Filters: English, Humans, from 2005 - 2025		
**Scopus (Elsevier) Coverage: From database inception-2026-01-15**	(TITLE-ABS-KEY ("Medical Faculty") OR TITLE-ABS-KEY ("Medical Faculties") OR TITLE-ABS-KEY ("Academic Medical Faculty") OR TITLE-ABS-KEY ("Clinical Faculty") OR TITLE-ABS-KEY ("Medical Educator*") OR TITLE-ABS-KEY ("Medical Teacher*") OR TITLE-ABS-KEY ("Medical Instructor*") OR TITLE-ABS-KEY ("Health Professions Faculty") OR TITLE-ABS-KEY ("Academic Physician*") OR TITLE-ABS-KEY ("Physician Educator*") OR TITLE-ABS-KEY ("Clinical Educator*") OR TITLE-ABS-KEY ("Medical School Faculty") OR TITLE-ABS-KEY ("Academic Medicine Faculty") OR TITLE-ABS-KEY ("Basic Science Faculty") OR TITLE-ABS-KEY ("Preclinical Faculty") OR TITLE-ABS-KEY ("Medical Education Faculty")) AND (TITLE-ABS-KEY ("Educational Need*") OR TITLE-ABS-KEY ("Training Need*") OR TITLE-ABS-KEY ("Professional Need*") OR TITLE-ABS-KEY ("Development Need*") OR TITLE-ABS-KEY ("Learning Need*") OR TITLE-ABS-KEY ("Faculty Need*") OR TITLE-ABS-KEY ("Gap Analysis") OR TITLE-ABS-KEY ("Training Gap*") OR TITLE-ABS-KEY ("Educational Gap*") OR TITLE-ABS-KEY ("Competency Gap*") OR TITLE-ABS-KEY ("Skill* Gap*") OR TITLE-ABS-KEY ("Needs Identification") OR TITLE-ABS-KEY ("Needs Prioritization") OR TITLE-ABS-KEY ("Priority Setting") OR TITLE-ABS-KEY ("Needs Analysis") OR TITLE-ABS-KEY ("Training Requirement*") OR TITLE-ABS-KEY ("Educational Requirement*") OR TITLE-ABS-KEY ("Competency Assessment") OR TITLE-ABS-KEY ("Skills Assessment") OR TITLE-ABS-KEY ("Professional Development Need*") OR TITLE-ABS-KEY ("Needs Assessment")) AND (TITLE-ABS-KEY ("Faculty Development") OR TITLE-ABS-KEY ("Staff Development") OR TITLE-ABS-KEY ("Professional Development") OR TITLE-ABS-KEY ("Human Resources Development") OR TITLE-ABS-KEY ("Academic Development") OR TITLE-ABS-KEY ("Capacity Building") OR TITLE-ABS-KEY ("Faculty Training") OR TITLE-ABS-KEY ("Academic Staff Development") OR TITLE-ABS-KEY ("Continuing Medical Education") OR TITLE-ABS-KEY ("Professional Growth") OR TITLE-ABS-KEY ("Continuing Professional Development") OR TITLE-ABS-KEY ("Educational Development") OR TITLE-ABS-	60 results	All search terms are searched in the fields: “title”, “abstract” and “keywords” (here marked with “TITLE-ABS-KEY”) filters or limitations English language, articles ,Years 2005 - 2025

	KEY ("Teaching Development") OR TITLE-ABS-KEY ("Academic Capacity Building")) AND PUBYEAR > 2004 AND PUBYEAR < 2026 AND (LIMIT-TO (SUBJAREA , "MEDI") OR LIMIT-TO (SUBJAREA , "HEAL")) AND (LIMIT-TO (DOCTYPE , "ar")) AND (LIMIT-TO (LANGUAGE , "English")) AND (LIMIT-TO (SRCTYPE , "j"))		
**Web of Science-Core Collection (Clarivate) Coverage:** **From database inception-2026-01-15**	(((((((((((((((TS=("Medical Faculty")) OR TS=("Medical Faculties")) OR TS=("Academic Medical Faculty")) OR TS=("Clinical Faculty")) OR TS=("Medical Educator*")) OR TS=("Medical Teacher*")) OR TS=("Medical Instructor*")) OR TS=("Health Professions Faculty")) OR TS=("Academic Physician*")) OR TS=("Physician Educator*")) OR TS=("Clinical Educator*")) OR TS=("Medical School Faculty")) OR TS=("Academic Medicine Faculty")) OR TS=("Basic Science Faculty")) OR TS=("Preclinical Faculty")) OR TS=("Medical Education Faculty")AND(((((((((((((((((((((TS=("Needs Assessment")) OR TS=("Educational Needs Assessment")) OR TS=("Educational Need*")) OR TS=("Training Need*")) OR TS=("Professional Need*")) OR TS=("Development Need*")) OR TS=("Learning Need*")) OR TS=("Faculty Need*")) OR TS=("Gap Analysis")) OR TS=("Training Gap*")) OR TS=("Educational Gap*")) OR TS=("Competency Gap*")) OR TS=("Skill* Gap*")) OR TS=("Needs Identification")) OR TS=("Needs Prioritization")) OR TS=("Priority Setting")) OR TS=("Needs Analysis")) OR TS=("Training Requirement*")) OR TS=("Educational Requirement*")) OR TS=("Competency Assessment")) OR TS=("Skills Assessment")) OR TS=("Professional Development Need*") AND(((((((((((((TS=("Faculty Development")) OR TS=("Staff Development")) OR TS=("Professional Development")) OR TS=("Human Resources Development")) OR TS=("Academic Development")) OR TS=("Capacity Building")) OR TS=("Faculty Training")) OR TS=("Academic Staff Development")) OR TS=("Continuing Medical Education")) OR TS=("Professional Growth")) OR TS=("Continuing Professional Development")) OR TS=("Educational Development")) OR TS=("Teaching Development")) OR TS=("Academic Capacity Building")<br>#3 AND #2 AND<br>#1 and 2005 or 2025 or 2024 or 2023 or 2022 or 2021 or 2020 or 2019 or 2018 or 2017 or 2016 or 2015 or 2014 or 2013 or 2012 or 2011 or 2010 or 2009 or 2008 or 2007 or 2006 or 2005 (Publication Years) and Article (Document Types) and English (Languages)	77 results	All search terms are searched in the field: “Topic” (including title, abstract and author supplied keywords, here marked with “TOPIC”). filters or limitations English language, articles ,<br>Years 2005 - 2025

	https://www.webofscience.com/wos/woscc/summary/59bcf6a3-caf3-435c-98ee-3e47c85d4613-0198adb328/relevance/1			
**Total no. references identified**		262		
**Total no. unique references identified after automatic de-duplication in Covidence**		193		
**Duplicate**		69		
