# Supplementary material for: Mapping faculty development needs in medical education: a bibliometric analysis
Source: Front Med (Lausanne). 2026 Jul 1;13:1858624. doi: 10.3389/fmed.2026.1858624 (PMC13370283; doi:10.3389/fmed.2026.1858624)
Supplement: Supplementary file 2 [file Data_Sheet_2.PDF]

Supplementary Material 2

Synonym and Exclusion List Used for Keyword Harmonization

This supplementary file presents the synonym and exclusion list used in the keyword analysis. In Table S2.1, the preferred term is the term retained in the analysis, while the listed variants were merged with it. Table S2.2 lists the terms excluded from the analysis because they reflected generic demographic labels, geographic identifiers, publication or metadata items, or topics outside the scope of the study.

Terms are separated by semicolons.

Table S2.1. Synonym groups used for keyword harmonization

Preferred term	Variants merged into the preferred term
**Needs assessment**	needs assessment; need assessment; needs-assessment; needs assessment survey; educational needs' assessment
**Faculty development**	faculty development; faculty-development; faculty-development program; faculty-development opportunity
**Staff development**	staff development; staff development/methods; staff development/methods/organization & administration; staff development/economics/methods/organization & administration; unit staff development
**Medical education**	medical education; medical-education; education medical
**Graduate medical education**	education medical graduate; education medical graduate/methods
**Undergraduate medical education**	education medical undergraduate; undergraduate medical education; undergraduate medical-education
**Continuing medical education**	education medical continuing; continuing medical education; continuing medical-education; continuing professional development; continuing professional-development; cpd
**Survey**	survey; surveys and questionnaires; questionnaire; questionnaires; national-survey
**Student**	student; students
**Medical students**	students medical; medical students; medical-students
**Teaching**	teaching; teaching methods; teaching/methods; teaching effectiveness; teaching skills; teaching round; teaching rounds; teaching and learning
**Medical faculty**	faculty medical; faculty medical/education; faculty medical/organization & administration; faculty medical/standards; faculty medical/statistics & numerical data
**Clinical competence**	clinical competence; clinical competence/standards; clinical competence/statistics & numerical data

Preferred term	Variants merged into the preferred term
**Competency-based medical education**	competency-based education; competency-based education/methods/standards; competency-based medical education
**Program development**	program development; program development/methods; program development/standards; development program; development programs
**Program evaluation**	program evaluation; program evaluation/methods; program evaluation/standards
**Health care**	health care; health-care; health care delivery; health care personnel; health care planning; health care quality
**Internship and residency**	internship and residency; internship and residency/methods; internship and residency/organization & administration; internship and residency/statistics & numerical data
**Professional competence**	professional competence; professional competence/standards; competence; competences
**Mentoring**	mentoring; mentorship in medical education; mentors; mentors/education; mentors/psychology
**Academic medical centers**	academic medical centers; academic medical-centers; academic medical centers/organization & administration; academic medical centers/standards
**Continuing education**	continuing education; education continuing
**Cross-sectional studies**	cross-sectional studies; cross-sectional study
**Retrospective studies**	retrospective studies; retrospective study
**Mixed methods**	mixed methods; mixed methods research
**Problem-based learning**	problem-based learning; problem-based learning/methods
**United States**	united states; united-states; southeastern united states
**Internal medicine**	internal medicine; internal-medicine; internal medicine residents
**Nursing education**	nursing education; nursing-education; education nursing
**Evidence-based medicine**	evidence-based medicine; evidence based medicine
**Clinical teacher**	clinical teacher; clinical teachers; clinical educator; clinical educators

Table S2.2. Terms excluded from the analysis

Category	Excluded terms
**Demographic descriptors**	female; male; adult; aged; middle aged; aged 80 and over; adolescent; child; young adult; sex factors; women; humans; human; normal human; east asian people; immigrant; race
**Geographic locations**	united states; united-states; canada; united kingdom; england; wales; europe; asia; germany; australia; austria; israel; mexico; chile; pakistan; indonesia; singapore; republic of korea; portugal; sweden; switzerland; turkey; vietnam; tanzania; rwanda; saudi arabia; baltimore; boston; california; colorado; iowa; texas; oregon; washington; middle east; papua; lesotho; china; africa; southeastern united states
**Publication and metadata terms**	article; priority journal; periodicals as topic; publishing; na; notreported; categories; centers; departments; part; guide; tips; tool; toolkit; lexicon; terminology as topic; dictionaries medical; vocabulary; vocabulary controlled; electronic address items; email strings such as @ items; corresponding author items such as A (CORRESPONDING AUTHOR)
**Clinical and non-target topics**	analgesics; analgesics opioid; opiate; opioid; opiate addiction; opioid-related disorders; chronic pain; pregnancy; hematology; hearing impairment; visual impairment; sleep disorder; asperger syndrome/diagnosis/epidemiology; sars-cov-2/isolation & purification; covid-19; covid-19/diagnosis/epidemiology/virology; musculoskeletal disease; musculoskeletal diseases; cardiovascular surgical procedures; cardiac catheterization; physical examination; injuries; earthquake
**Health service and care-delivery terms**	delivery of health care; patient care; patient safety; referral and consultation; outpatient; outpatients; health service; health services needs and demand; ambulatory care; tertiary care centers; primary health care; primary health care/standards; health care planning; health care survey; health care surveys; health centers; national health service; united states department of veterans affairs
**Personnel and administrative terms**	personnel; personnel management; personnel selection/methods; employee performance appraisal; manpower; personnel staffing and scheduling; private sector; public sector; business of medicine; business competencies
