# Supplementary material for: Mapping faculty development needs in medical education: a bibliometric analysis
Source: Front Med (Lausanne). 2026 Jul 1;13:1858624. doi: 10.3389/fmed.2026.1858624 (PMC13370283; doi:10.3389/fmed.2026.1858624)

SUPPLEMENTARY-FIGURES:

Figure S1. Country Production Over Time
Temporal evolution of research productivity across countries, illustrating how national contributions to faculty development research have developed throughout the study period.

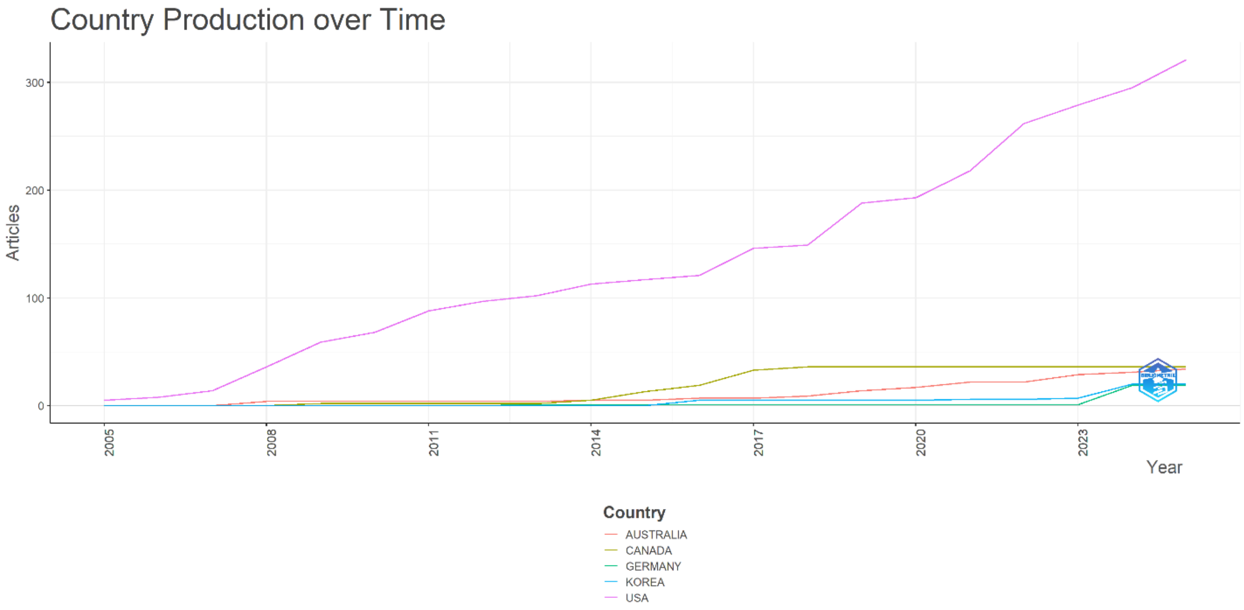

Figure S2. Authors' Production Over Time
Temporal distribution of publications by leading authors, illustrating productivity patterns across the study period.

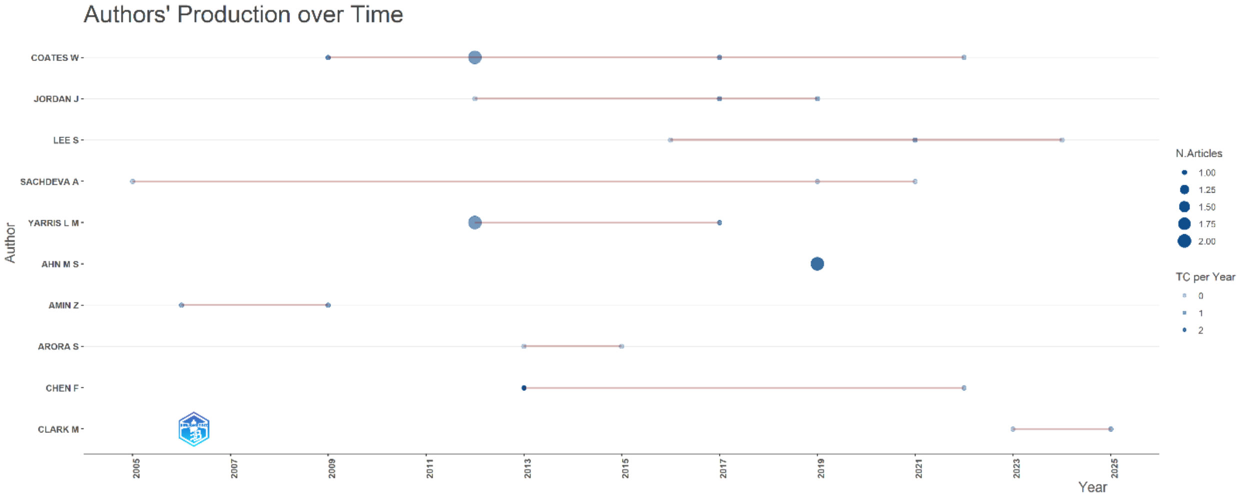

Figure S3. Source Impact (H-index of Journals)
Local citation impact of journals measured by H-index, indicating the influence of publication sources within the dataset.

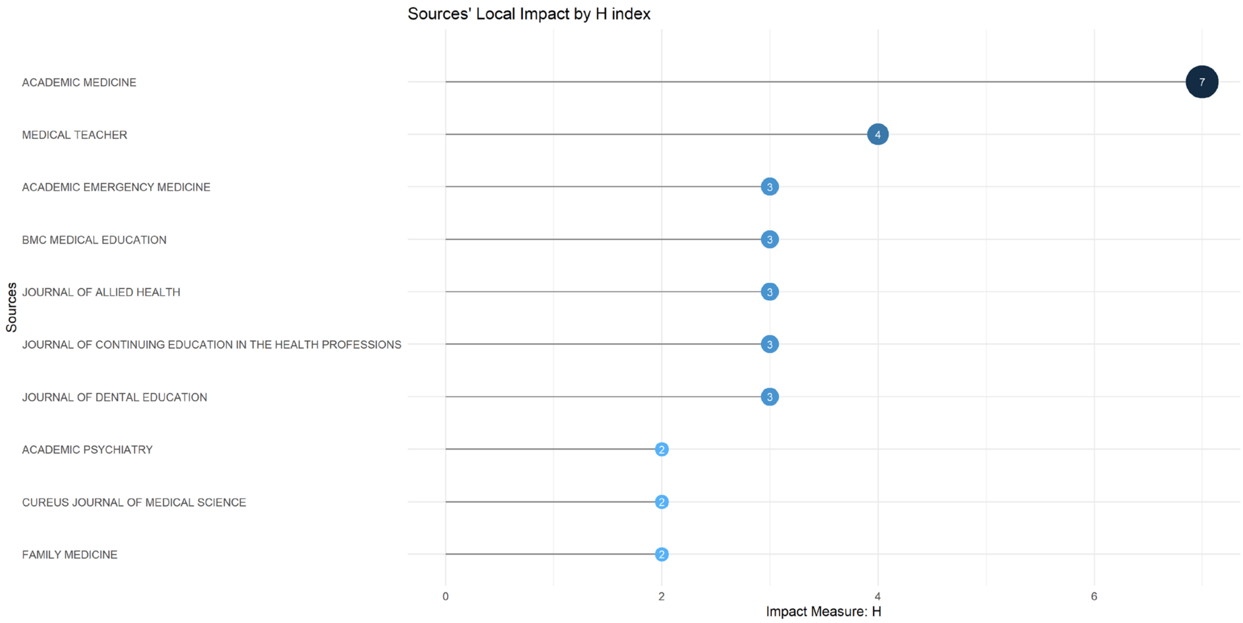

Figure S4. Most Relevant Affiliations
Institutional contributors with the highest number of publications in faculty development research.

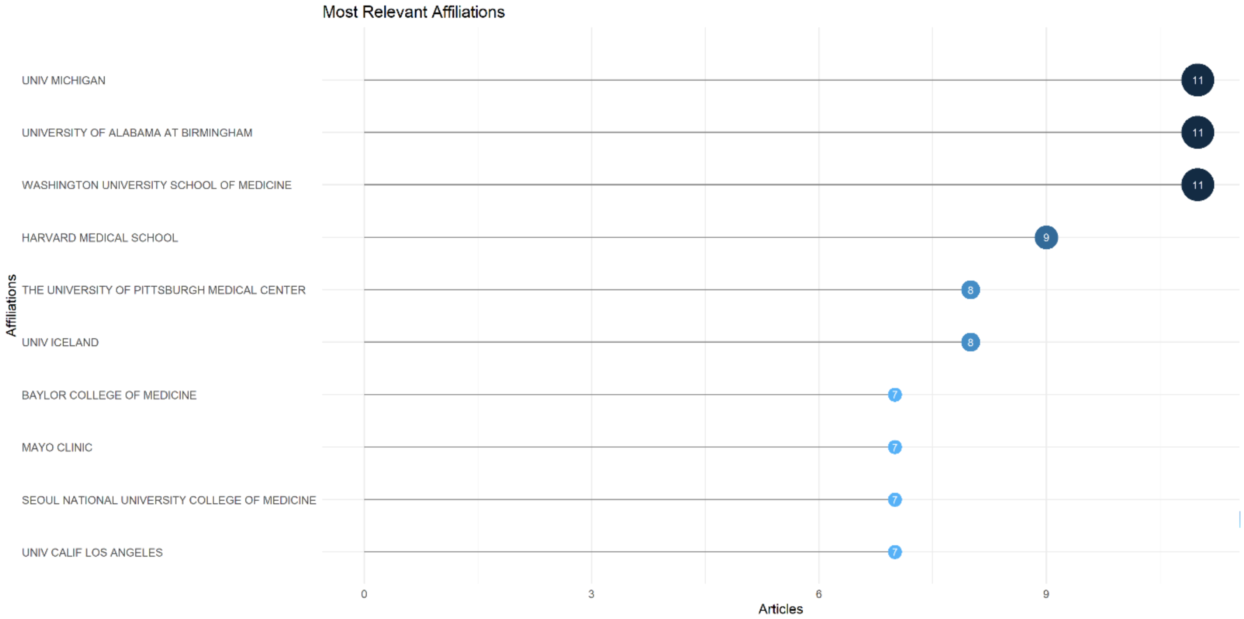

FigureS5. Sources' Production Over Time

Cumulative number of publications over time for leading journals in faculty development research, illustrating how major sources such as *Academic Medicine*, *BMC Medical Education*, and *Medical Teacher* have contributed to the growth of the literature.

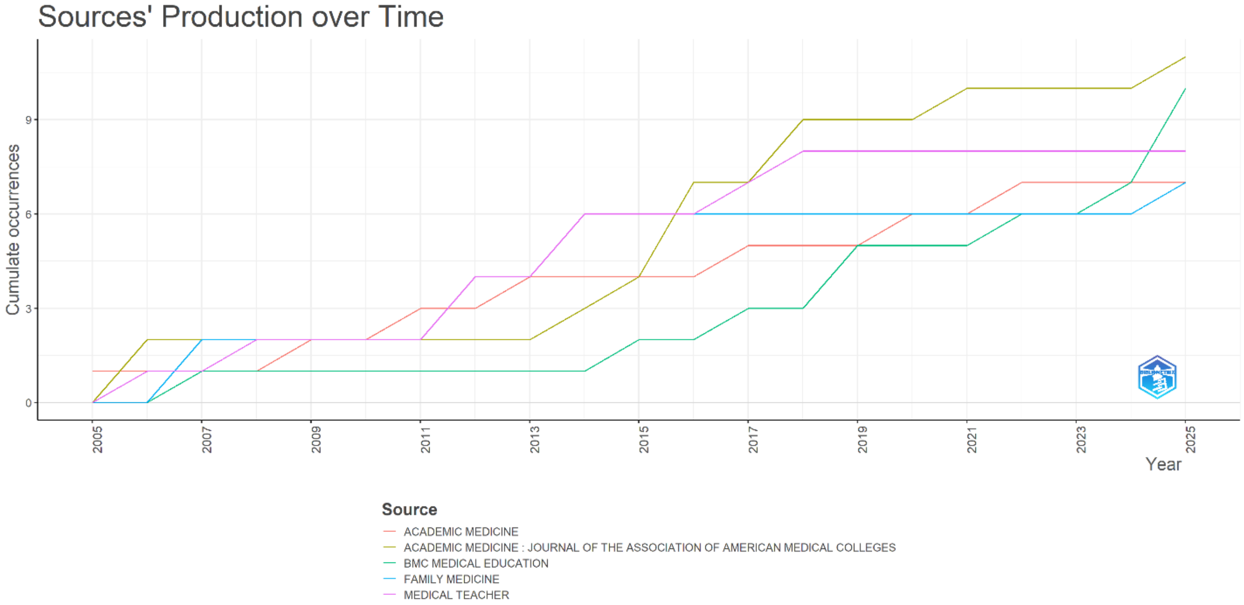

Figure S6. Most Relevant Words

Frequency distribution of the most commonly occurring keywords in the dataset, highlighting dominant concepts and research topics within the faculty development literature.

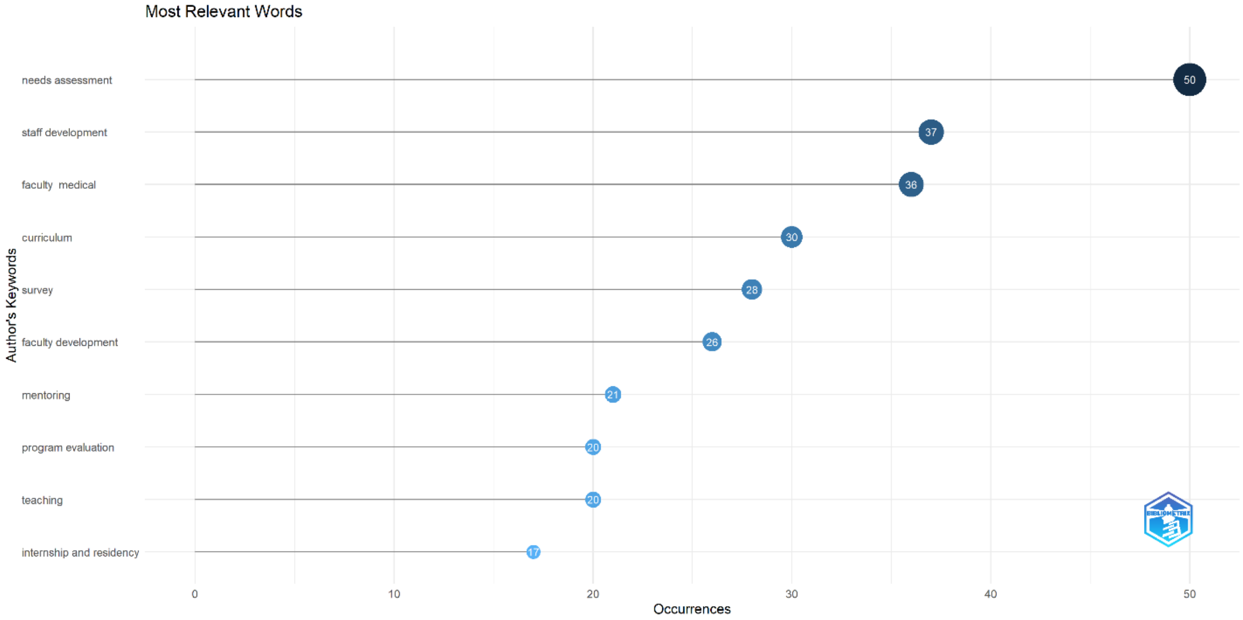

Figure S7. Word Cloud of Keyword Frequency
Visual representation of keyword frequency in faculty development research, where larger words indicate higher occurrence within the analysed publications.

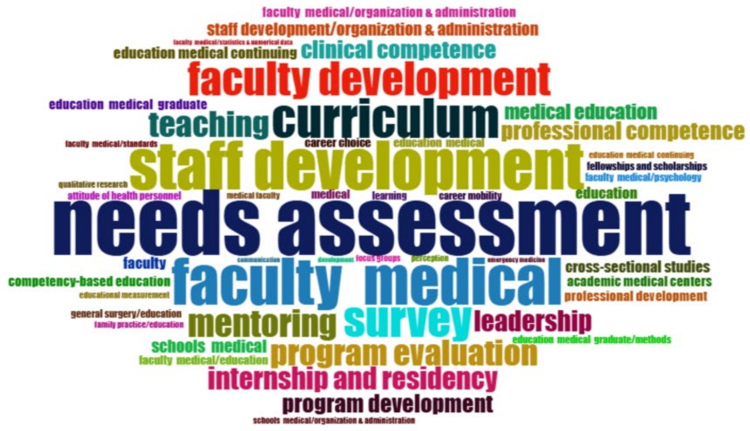

Figure S8. Temporal Trends of Research Topics in Medical Faculty Development (2005–2025)
Trend topics analysis showing the time span and relative frequency of keywords over the study period. Larger bubbles indicate higher keyword frequency.

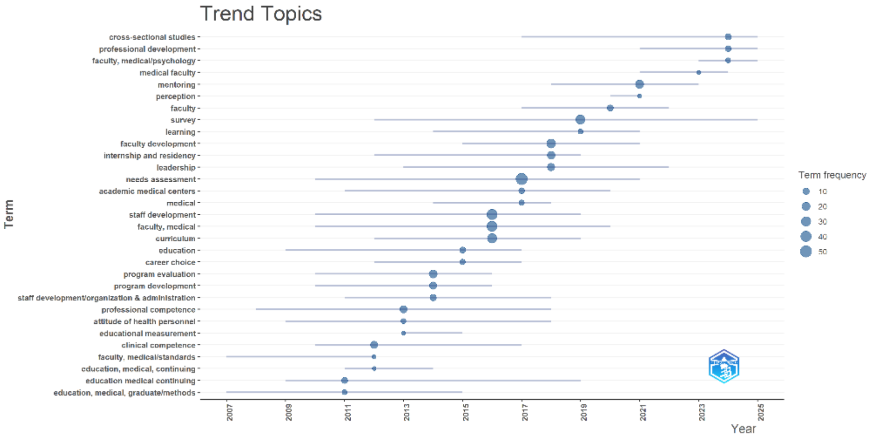

Figure S9. Cumulative Frequency of Keywords Over Time
Line graph showing cumulative occurrences of key research terms from 2005 to 2025, illustrating the growth of major topics in medical faculty development literature.

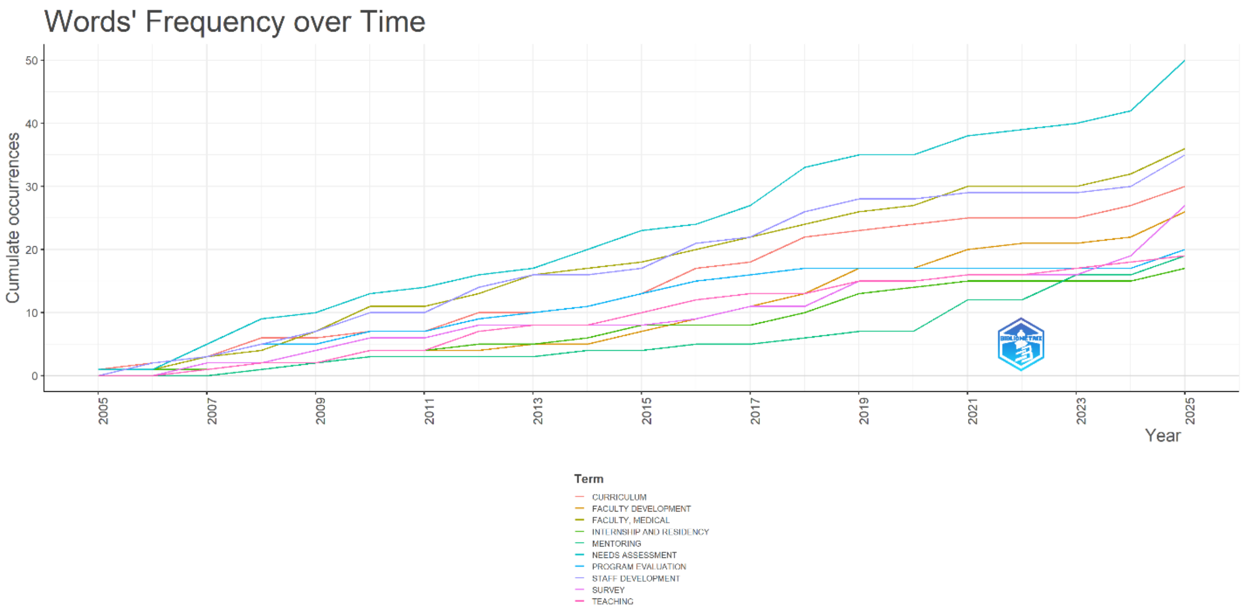

Supplement: Supplementary file 3 [file Data_Sheet_3.PDF]
